# Mitochondrial dysfunction in Wilson disease: a systematic review and meta–analysis across human and animal models

**DOI:** 10.3389/fmolb.2025.1712573

**Published:** 2025-12-17

**Authors:** Raya Amin, Valentina Medici, Erik D. Fausak, Keren Sierra, Ieleen Li, Joshua Gong, Cecilia Giulivi

**Affiliations:** 1 Department of Molecular Biosciences, School of Veterinary Medicine, University of California, Davis, CA, United States; 2 Department of Internal Medicine, Division of Gastroenterology and Hepatology, University of California, Davis, CA, United States; 3 University Library, University of California, Davis, CA, United States; 4 Medical Investigations of Neurodevelopmental Disorders (M.I.N.D.) Institute, University of California, Davis, CA, United States

**Keywords:** copper toxicity, complex IV, citrate synthase, oxidative stress, MtDNA copy number, bioenergetics, liver metabolism, translational hepatology

## Abstract

**Background & Aims:**

Wilson disease (WD) is a genetic disorder of copper metabolism caused by *ATP7B* mutations, leading to hepatic and systemic copper accumulation. While lysosomes are early storage sites, mitochondria appear highly vulnerable to copper toxicity. We performed a systematic review and meta–analysis to assess mitochondrial outcomes in WD patients and animal models.

**Methods:**

PubMed, Scopus, and SciFinder were searched through 11 September 2025, for studies reporting hepatic mitochondrial endpoints in WD (in patients and models using mice, rats, and dogs). Outcomes included mitochondrial copper, morphology, oxidative stress, mtDNA copy number, ATP production, and respiratory Complex activities. Random–effects meta–analyses were conducted.

**Results:**

Thirteen studies met the inclusion criteria. Mitochondrial copper was consistently elevated (standardized mean difference ±standard error: 6.7 ± 0.9, P < 0.001), with ultrastructural abnormalities (4 ± 2, P = 0.012). Oxidative stress markers increased (2.9 ± 0.9, P = 0.001), while MnSOD and aconitase declined with disease progression. mtDNA copy number was reduced (−0.7 ± 0.3, P = 0.032). ATP synthesis (−1.5 ± 0.6, P = 0.023) and Complex activities (−1.0 ± 0.3, P = 0.001) were impaired, especially in older or symptomatic subjects. Citrate synthase activity increased (2.8 ± 0.9, P = 0.003), consistent with compensatory biogenesis. Several abnormalities appeared in presymptomatic or young animals.

**Conclusion:**

Across human and animal studies, hepatic mitochondria in WD exhibit copper accumulation, structural injury, impaired bioenergetics, oxidative stress, and mitochondrial genome loss. Mitochondrial dysfunction arises early and worsens with progression, highlighting it as a central pathogenic feature and therapeutic target.

## Highlights

- Mitochondrial copper overload is an early and consistent feature of Wilson disease (WD) in both patients and animal models.
- Hepatic mitochondria show structural abnormalities and progressive bioenergetic failure, with reduced ATP production and a lower mitochondrial genome, especially in older or symptomatic subjects.
- Oxidative stress is elevated, while antioxidant defenses decline with age and disease progression.
- Increased citrate synthase activity suggests compensatory mitochondrial biogenesis but appears insufficient to prevent damage.
- Evidence from one study suggests that enhanced autophagy and mitochondria–lysosome crosstalk may serve as protective mechanisms against copper toxicity in WD.
- Meta–analysis underscores mitochondrial dysfunction as central to WD and highlights mitochondria–lysosome crosstalk as a therapeutic target.

## Introduction

Copper is an essential trace element involved in numerous physiological processes, including neurotransmitter synthesis, collagen formation, growth, wound healing, and angiogenesis (Gromadzka et al., 2024; Festa and Thiele, 2011). Within mitochondria, copper plays a crucial role in ATP production, serving as a cofactor for cytochrome *c* oxidase, a key enzyme in the electron transport chain (Gromadzka et al., 2024; Timon-Gomez et al., 2018; Ruiz et al., 2021). Additionally, copper is integral to the antioxidant defense system, acting as a cofactor for Cu/Zn superoxide dismutase, which detoxifies reactive oxygen species (ROS) (Gromadzka et al., 2024; Johnson and Giulivi, 2005). Thus, copper plays a dual role in cellular energy metabolism and protection against oxidative damage.

However, when copper accumulates beyond physiological levels, it can become toxic. One proposed mechanism of copper toxicity involves the Fenton and Haber–Weiss reactions, leading to the generation of hydroxyl radicals and increased oxidative stress, which can damage cellular components such as proteins, lipids, and DNA (Gromadzka et al., 2024). Mitochondria are particularly susceptible to copper–induced damage due to their role in copper storage and utilization (Lal and Sourkes, 1971), as well as their involvement in ROS production (Gromadzka et al., 2024; Arc et al., 2005). Under such conditions, mitochondria exhibit significant structural and functional abnormalities, including cristae dilation, pleomorphism, inclusion bodies, and dense matrices (Gromadzka et al., 2024).

Wilson disease (WD) is an autosomal recessive disorder caused by mutations in the *ATP7B* gene, which impairs biliary copper excretion (Sanchez-Monteagudo et al., 2021) and leads to its progressive accumulation in the liver and brain (Gromadzka et al., 2024; Gerosa et al., 2019). In the liver, copper overload drives mitochondrial dysfunction and oxidative stress (Nagasaka et al., 2006), contributing to hepatocellular injury, cirrhosis, and, in severe cases, acute liver failure (Gromadzka et al., 2024; Gu et al., 2000; Zischka and Lichtmannegger, 2014). Evidence from patients and animal models demonstrates mitochondrial DNA (mtDNA) deletions (Medici et al., 2020; Mansouri et al., 1997), reduced oxidative phosphorylation (OXPHOS) (Gu et al., 2000; Sokol et al., 1993), and ultrastructural abnormalities, including cristae dilation and electron–dense inclusions (Zischka and Einer, 2018; Sokol et al., 1994; Roberts et al., 2008). Increased activity of mitochondrial manganese superoxide dismutase (Mn–SOD), a compensatory antioxidant response, has also been reported (Klein et al., 1998; Polishchuk et al., 2019).

Despite this body of evidence, the contribution of mitochondrial dysfunction to WD pathogenesis remains a topic of debate. Some studies implicate mitochondria as a central target of copper toxicity (Gu et al., 2000; Sokol et al., 1993; Sokol et al., 1994; Sternlieb, 1992), while others suggest greater involvement of alternative compartments, such as lysosomes (Gromadzka et al., 2024; Lal and Sourkes, 1971).

To investigate the role of mitochondria, several WD animal models have been established, including the toxic milk (*Atp7b* ^*tx/tx*^) mouse, the *Atp7b* knockout mouse (*Atp7b* ^−/−^), the Long–Evans Cinnamon (LEC) rat, the Long–Evans Agouti Piebald (LPP) rat, and murine substrains such as TX–J and TX–R (Table 1). Each model reproduces different aspects of the human condition: the toxic milk and TX substrains mimic missense mutations found in patients but are confounded by the “toxic milk” phenotype; knockout mice provide a clean loss–of–function system with consistent hepatic disease but limited genetic diversity; the LEC rat develops fulminant hepatitis and hepatocellular carcinoma but is affected by early lethality; similarly, in the LPP rat, which is derived from the LEC rat, rapid hepatic copper accumulation evolves into acute liver failure followed by mortality which affects almost all rats. Mitochondrial pathology has been reported in several WD models: LPP rats show copper–driven mitochondrial accumulation, structural lesions, reduced ATP, membrane depolarization, and oxidative stress (Zischka and Lichtmannegger, 2014; Zischka and Einer, 2018; Einer et al., 2019; Lichtmannegger et al., 2016; Zischka et al., 2011); LEC rats display mitochondrial copper accumulation, oxidative stress, membrane crosslinking, and increased susceptibility to permeability transition (Zischka et al., 2011; Li et al., 1991; Terada et al., 1999). Copper toxicosis in Bedlington terriers overlaps with the human Wilson disease phenotype. It is primarily associated with a recessive mutation in the *COMMD1* gene, also known as *MURR1*, an interacting ATP7B protein, which impairs hepatic copper excretion and leads to copper accumulation in the liver as observed in Wilson disease. This mutation was first identified in 2005 by Forman et al., who characterized a 39.7 kb deletion in exon 2 of the *COMMD1* gene on canine chromosome 10 (Forman et al., 2005). Subsequent studies have confirmed that this deletion is a major cause of copper toxicosis in Bedlington terriers (Spee et al., 2006; de Bie et al., 2007). However, some affected dogs lack the *COMMD1* deletion, suggesting that other genetic factors may also contribute to the disease. A 2023 study by Haywood et al. reported an association between copper toxicosis and a missense mutation in the *ATP7B* gene in Bedlington terriers, indicating a more direct potential overlap with WD mechanisms (Haywood et al., 2023). As in Wilson disease, Bedlington terriers exhibit mitochondrial oxidative damage and structural changes, although genetic characterization was not performed in the Sokol et al. (1994) cohort (Sokol et al., 1994).

**TABLE 1:** Comparison of Wilson disease models across species with reported mitochondrial findings.

Feature	Toxic milk (*Atp7b* ^tx/tx^)	ATP7B knockout (*Atp7b* ^−/−^)	TX–J mouse	TX–R mouse	Long–Evans cinnamon (LEC) rat	Long–Evans agouti piebald (LPP) rat	Dogs (bedlington terriers)	Human Wilson disease
Genetic alteration	Missense mutation in *Atp7b* (M1356V)	Complete *Atp7b* deletion	Same M1356V missense mutation as toxic milk	Same mutation as toxic milk, maintained as a substrain	Spontaneous deletion in *Atp7b* exon 8	Targeted *Atp7b* KO on Long–Evans agouti piebald background	Not genetically characterized in (17); commonly associated with *COMMD1* mutation in breed	>700 known mutations in *ATP7B* (mostly missense, some nonsense, insertions, deletions)
Origin	Naturally occurring mutant (1960s, Texas)	Engineered via targeted knockout	Derived from TX strain, bred at jackson laboratory	Derived from TX strain, bred at rockefeller university	Inbred Long–Evans strain (Japan, 1980s)	Derived from *Atp7b* KO on Long–Evans agouti piebald; widely studied in europe (zischka lab)	Bedlington terrier breed	Autosomal recessive disorder (∼1/30,000 worldwide)
Milk phenotype	Homozygous mutant females produce copper–deficient “toxic” milk; pups must be fostered	No toxic milk phenotype	Same toxic milk phenotype as TX	Same toxic milk phenotype as TX	No toxic milk phenotype	No toxic milk phenotype	Not applicable	Not applicable
Copper metabolism	Hepatic copper accumulation; defective copper incorporation into CP	Hepatic copper accumulation; no CP activity	Hepatic copper overload; impaired copper transport	Hepatic copper overload; impaired copper transport	Severe hepatic copper accumulation; very low/absent CP	Severe hepatic copper accumulation; mitochondrial copper overload in hepatocytes	Hepatic copper accumulation; normal CP	Hepatic and extrahepatic copper accumulation (liver, brain, kidney, cornea); low CP activity
Onset and severity	Variable, strain–dependent	Earlier and more consistent hepatic disease onset	Progressive hepatic disease; variable onset	Progressive hepatic disease; variable onset	Early fulminant hepatitis (2–4 months); often fatal	Early fulminant hepatitis (2–4 months); often fatal	Variable; develops copper toxicosis as young adults (breed–dependent)	Mostly childhood and early adulthood; highly variable onset (asymptomatic to acute liver failure)
Pathology	Hepatic copper overload, inflammation, fibrosis; some neurological features reported	Severe hepatic pathology (steatosis, hepatitis, fibrosis); less neurological involvement	Hepatic copper accumulation, inflammation, fibrosis; CNS features unclear	Hepatic copper accumulation, inflammation, fibrosis; CNS features unclear	Acute hepatitis, necrosis, hepatocellular carcinoma (if animals survive); neurological features poorly described	Hepatic copper overload; mitochondrial structural damage, functional defects. No reports on neurological features	Hepatic copper accumulation; mitochondrial oxidative damage and structural changes observed	Liver disease (steatosis, hepatitis, cirrhosis, acute liver failure); neurological symptoms (movement disorders, psychiatric manifestations); Kayser–Fleischer rings
Advantages	Mimics missense mutations seen in most patients; spontaneous model; widely studied	Consistent phenotype; mechanistic clarity from null mutation	Widely available, reproducible substrain; retains TX features	Alternative substrain for reproducibility; similar to TX	Strong hepatic WD phenotype; used in hepatocarcinogenesis studies	Strong hepatic WD phenotype; best characterized model for mitochondrial pathology	Non–rodent model; shows mitochondrial involvement and copper–driven oxidative damage	Captures full clinical spectrum (hepatic, neurological, psychiatric, ophthalmologic)
Disadvantages	Toxic milk complicates breeding; variable disease expression	Less representative of clinical diversity (few WD patients have null alleles)	Limited by toxic milk phenotype and breeding challenges	Same as TX and TX–J limitations	Early lethality limits long–term and extrahepatic studies	Requires specialized colonies; less widely available than LEC; limited neurological characterization	Only one study; genetic status unknown in (17); breed–specific; limited mechanistic detail	High heterogeneity in presentation; challenging for controlled mechanistic studies
Overall clinical relevance	Reflects genetic heterogeneity of WD (missense mutation)	Useful for mechanistic studies but less representative of most patients	Relevant for hepatic pathology; similar to TX	Useful for cross–validation; similar to TX	Represents severe hepatic WD; less useful for neurological/variable forms	Highly relevant for hepatic pathology and mitochondrial dysfunction, closely parallels human hepatic WD mitochondrial lesions	Illustrates copper–driven mitochondrial pathology in a large animal model; limited mechanistic insight due to genetic uncertainty	Gold standard; defines the full disease spectrum and guides translational relevance

Abbreviations: CP, ceruloplasmin; CNS, central nervous system.

All the animal models listed in Table 1 are characterized by minimal or no neurological involvement. Human WD, by contrast, is genetically and clinically heterogeneous, with both hepatic and neurological presentations.

While these models have yielded important insights, findings are often heterogeneous and sometimes contradictory, reflecting differences in study design, outcome measures, and the intrinsic limitations of each system. Human studies remain definitive but are hampered by small cohorts, tissue accessibility, and clinical variability. By quantitatively integrating data from all models—including patients—through systematic review and meta–analysis, we aimed to overcome these individual limitations, reduce bias, and provide a more comprehensive understanding of the role of mitochondrial dysfunction in the pathogenesis of WD.

## Methods

### Search strategy

A systematic literature search was performed in PubMed, Scopus, and SciFinder to identify studies reporting mitochondrial dysfunction in WD. The PubMed search string was: (Wilson disease OR Wilson’s disease OR copper toxicity) AND (mitochondrial OR mitochondria OR mitochondrion OR oxidative stress OR free radic*), with filters set to “journal article” and “English.” See the Supplementary Information for details on the search strategy. Equivalent terms and filters were used in SciFinder and Scopus. Searches included all records published up to 11 September 2025.

### Eligibility criteria

Studies were eligible if they were original research articles reporting quantitative outcomes related to mitochondrial function in WD patients or animal models, with data available for both WD and wild–type (WT) or other appropriate controls. No restrictions were placed on animal species or tissue type. Exclusion criteria included: reviews, editorials, commentaries, proceedings, case reports, non–English publications, cell line–only studies, and studies lacking relevant control groups.

### Study selection

All retrieved records (n = 11,673) were uploaded to Covidence (Veritas Health Innovation, Melbourne, Australia) for screening by at least two reviewers. After removing 6,599 duplicates, 5,074 unique titles and abstracts were screened. One hundred and twelve articles were selected for full–text review, of which 79 were excluded for irrelevance (wrong design), leaving 33 potentially eligible studies. The majority focused on liver mitochondria, although a small number investigated other tissues (e.g., brain lactate by MRI in WD patients, testes in TX mice, colon carcinoma Caco–2 cells). Since 30 of 33 studies focused on the liver (including one with data on liver and PBMC), subsequent analyses were limited to this organ.

**FIGURE 1:**
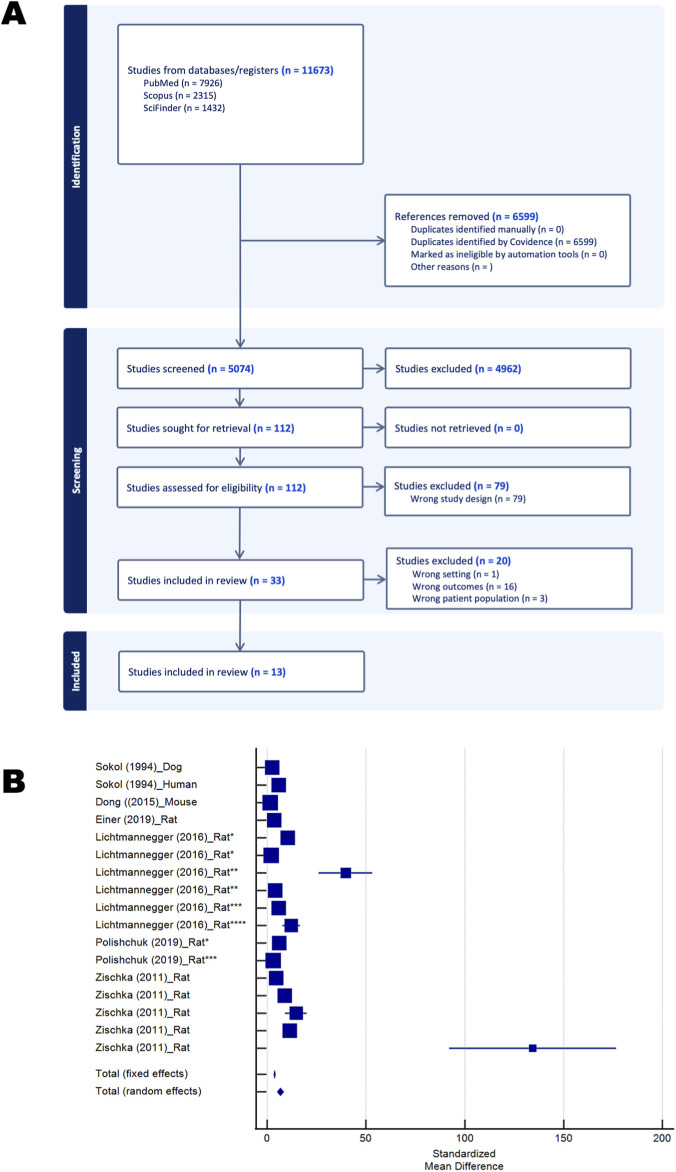
PRISMA flow diagram of study selection and forest plot of mitochondrial copper outcomes in Wilson disease and models. **(A)** PRISMA 2020 flow diagram showing identification, screening, eligibility, and inclusion of studies reporting mitochondrial outcomes in Wilson disease (generated in Covidence). **(B)** Forest plot of standardized mean differences (SMD, Hedges’ *g*) of mitochondrial copper levels produced in MedCalc. Individual studies are represented by markers at their central SMD values, with horizontal lines indicating 95% confidence intervals (CIs). Marker size reflects the relative weight of each study, which differs under the fixed–effects and random-effects models. Between–study heterogeneity was accounted for in the random–effects analysis using the DerSimonian–Laird method. Two pooled estimates are displayed at the bottom of the plot: the fixed–effects summary and the random–effects summary, each represented by a diamond with its width corresponding to the 95% CI. Studies are labeled by first author, year, and species. In the Lichtmannegger study, one to four asterisks indicate disease stage (affected, onset, diseased, moribund), while in the Polishcuk study, asterisks denote affected (*) and diseased (**) groups.

At full–text review, an additional 17 studies were excluded due to methodological limitations (e.g., absence of quantitative mitochondrial outcomes, mitochondrial copper content only, protein expression without corresponding activity, use of crude mitochondrial fractions, lack of appropriate controls, single control sample, treatment–only studies, or unsuitable format). Ultimately, 13 studies met the inclusion criteria for quantitative synthesis (Nagasaka et al., 2006; Gu et al., 2000; Medici et al., 2020; Mansouri et al., 1997; Sokol et al., 1994; Roberts et al., 2008; Polishchuk et al., 2019; Einer et al., 2019; Lichtmannegger et al., 2016; Zischka et al., 2011; Dong et al., 2015; Ohhira et al., 1995; Sauer et al., 2011) (Figure 1A; Table 2).

**TABLE 2:** Characteristics of Studies Included in the Systematic Review and Meta–Analysis of Mitochondrial Dysfunction in Wilson disease.

Study	Species/Model	Tissue	Key findings
Gu et al. (2000)	Human WD	Liver	OXPHOS defects, reduced Complex I–V activity
Mansouri et al. (1997)	Human WD	Liver	Premature mtDNA oxidative aging
Medici et al. (2020)	Human WD/Mouse KO	PBMC/Liver	mtDNA depletion–like syndrome, reduced Complex IV
Nagasaka et al. (2006)	Human WD	Liver	Elevated ROS, age–related antioxidant decline
Polishchuk et al. (2019)	Human WD	Liver	Autophagy protects hepatocytes from copper toxicity
Sokol et al. (1994)	Human WD/Bedlington terriers	Liver	Oxidative mitochondrial injury
Dong et al. (2015)	Mouse KO	Liver	Neuronal damage despite low copper; mitochondrial vulnerability
Roberts et al. (2008)	Mouse tx–j	Liver	Structural abnormalities, impaired ATP production
Sauer et al. (2011)	Mouse KO	Liver	Severe mitochondrial dysfunction, altered cholesterol metabolism
Einer et al. (2019)	Rat LPP	Liver	High–calorie diet worsens mitochondrial dysfunction and liver injury
Lichtmannegger et al. (2016)	Rat LPP	Liver	Methanobactin reverses acute liver failure
Ohhira et al. (1995)	Rat LEC	Liver	Altered antioxidant enzymes, increased lipid peroxidation
Zischka et al. (2011)	Rat LPP/LEC	Liver	Mitochondrial membrane crosslinking and destruction

### Data extraction

At least two reviewers independently extracted data using a standardized template, with disagreements resolved by consensus. Extracted information included: first author, year of publication, study title, species (strain/breed), sex and age of participants, organ studied, model or patient cohort, sample size per group, and reported mitochondrial outcomes (mean ± SD or frequency).

### Outcomes of interest

Mitochondrial outcomes were classified into six categories: (1) mitochondrial copper content; (2) oxidative stress markers; (3) mtDNA copy number; (4) oxygen uptake–linked ATP production; (5) enzymatic activities of Complexes (including citrate synthase); and (6) mitochondrial morphology.

### Risk of bias assessment

The risk of bias was assessed qualitatively based on the study design, adequacy of the control group, transparency of reporting, and methodological rigor. Newcastle–Ottawa quality assessment scale (NOS) was used for case–control studies (Wells et al., 2025). Inconsistencies were discussed to reach a consensus, and a third researcher was available for discussion in case consensus could not be reached by the two primary researchers. Studies lacking quantitative mitochondrial outcomes or appropriate controls were excluded before synthesis.

### Reporting bias assessment

Although publication bias was formally assessed using funnel plots and regression–based methods (MedCalc), the relatively small number of included studies (n = 13 total and <13/outcome) limited the reliability of these assessments. Consequently, potential bias due to selective reporting or unpublished negative findings was not further included, as the pooled estimates should be interpreted with caution.

### GRADE assessment

The *c*ertainty of evidence for each outcome was assessed using the GRADE (Grading of Recommendations Assessment, Development and Evaluation) framework. This approach evaluates the confidence in the effect estimates based on five domains: risk of bias, inconsistency, indirectness, imprecision, and publication bias. For each outcome, we considered study design, sample size, species/model relevance, effect magnitude, and heterogeneity (I^2^). Outcomes were classified as high, moderate, low, or very low certainty, with downgrades applied when studies demonstrated substantial limitations, high heterogeneity, small sample sizes, or indirect evidence. The results of this assessment are summarized in Table 3.

**TABLE 3:** GRADE assessment of outcomes in Wilson disease meta–analysis.

Outcome category	Outcome	n Studies (species)	SMD ±SE (95% CI)	I^2^ (%)	Certainty (GRADE)	Rationale
Copper accumulation	Mitochondrial copper content	6 (human, dog, mouse, rat)	6.7 ± 0.9 (5.00–8.38)	92.6	Moderate	Large effect; high heterogeneity; mixed species reduces certainty
Structural changes	Aberrant mitochondria	2 (rat)	4 ± 2 (0.98–7.30)	82.6	Low	Few studies, single species, high heterogeneity
Oxidative stress	TBARS, mtROS, conjugated dienes	3 (human, dog, mouse, rat)	2.9 ± 0.9 (1.23–4.74)	89.2	Moderate	Significant effect, but high heterogeneity and limited sample sizes
	MnSOD and aconitase activities	4 (human, mouse, rat)	0.1 ± 0.5 (−0.86– 1.15)	87.2	Low	Non–significant, high heterogeneity, wide CI
Genomic integrity	mtDNA copy number	2 (human, mouse KO and TX–J)	−0.7 ± 0.3 (−1.42 to −0.06)	66.8	Low	Few studies, single tissue types, moderate heterogeneity
Bioenergetics	Oxygen–linked ATP production	2 (mouse, rat)	−1.5 ± 0.6 (−2.76 to −0.21)	87.0	Low	Small number of studies, high heterogeneity
	Complex activities (overall)	6 (human, mouse KO and TX–J, rat)	−0.6 ± 0.3 (−1.12 to −0.13)	86.2	Moderate	Significant overall reduction; high heterogeneity and mixed species
	Complex I activity	3 (human, mouse KO, rat)	0.0 ± 0.7 (−1.31– 1.28)	84.6	Very low	Non–significant, high heterogeneity
	Complex II activity	2 (mouse KO, rat)	−0.9 ± 0.5 (−1.92– 0.16)	83.5	Low	Non–significant, high heterogeneity
	Complex II–III activity	2 (mouse tx–j, human)	0.0 ± 0.6 (−1.19– 1.26)	83.5	Low	Non–significant, high heterogeneity
	Complex III activity	1 (mouse KO)	−0.5 ± 0.3 (−1.10–0.09)	0	Very low	Single study; moderate effect; very low certainty
	Complex IV activity	4 (mouse KO and TX–J, rat)	−1.4 ± 0.5 (−2.48 to −0.38)	89.6	Moderate	Significant reduction; high heterogeneity
	Complex V activity	2 (mouse KO, rat)	−0.7 ± 0.3 (−1.32–0.02)	31.1	Low	Borderline significance; low study number
	Citrate synthase activity	3 (human, mouse KO and TX–J)	0.7 ± 0.9 (−1.17– 2.47)	92.4	Low	Non–significant, very high heterogeneity
	Citrate synthase activity (adults only)	3 (human, mouse KO and TX–J)	2.8 ± 0.9 (0.98–4.57)	87.4	Moderate	Significant effect; high heterogeneity

SMD, standardized mean difference; SE, standard error; CI, confidence interval; I^2^, between–study heterogeneity. Positive SMD, indicates an increase in WD, models relative to controls; negative SMD, indicates a decrease. Detailed forest plots and study–level data are provided in the Figures and Supplementary Information. Abbreviations: thiobarbituric acid–reactive substances (TBARS).

### Data synthesis and statistical analysis

Meta–analyses were performed using MedCalc® Statistical Software version 23.3.2 (MedCalc Software Ltd., Ostend, Belgium; https://www.medcalc.org; 2025). Study weights were assigned as the inverse of the standard error, favoring larger and more precise studies. Both fixed–effects and random–effects models were applied, with the latter selected when heterogeneity was present. Between–study heterogeneity was assessed with Cochran’s Q test (P < 0.10 considered significant) and the I^2^ statistic, where values of 30%–60% indicated moderate and >50% substantial heterogeneity (Higgins et al., 2022). Publication bias was evaluated using Egger’s regression test and Begg’s rank correlation test. Forest plots were generated to display study–specific effect sizes and 95% confidence intervals (CIs), with marker size proportional to study weight. The pooled effect was represented by a diamond, with its center indicating the summary estimate and its width the 95% CI. Funnel plots were used to assess asymmetry. A two–sided P value <0.05 was considered statistically significant.

### Hepatic lactate, glucose, and pyruvate levels in *Atp7b* ^−/−^ mice

All animal studies were conducted in accordance with the ethical standards of the American Association for Accreditation of Laboratory Animal Care (AAALAC). Protocols were reviewed and approved annually by the University of California, Davis Institutional Animal Care and Use Committee (IACUC). Humane care and handling of animals followed the *Guide for the Care and Use of Laboratory Animals*, published by the National Research Council (National Institutes of Health publication 86–23, revised 1985). Experiments were performed using wild–type C3HeB/FeJ (C3H) mice and the Jackson Laboratory toxic milk mouse model of Wilson disease, *C3He*–*Atp7b* ^*tx-J/J*^ (tx–j). Breeding colonies were maintained under controlled environmental conditions (20 °C–23 °C, 45%–65% relative humidity, 14:10 h light/dark cycle). C3H mice were provided with standard LabDiet chow (Purina Mills, catalog #5001) and served as controls, whereas tx–j mice received a purified AIN–76A diet (Dyets Inc., catalog #D110098). For each experimental group, 20 mice were included (10 males and 10 females). Because tx–j females produce copper-deficient milk that is inadequate for supporting neonatal growth beyond approximately postnatal day 10, tx–j litters were cross–fostered to lactating C3H dams by postnatal day 7. Offspring were weaned at 3 weeks of age, and livers were harvested from both groups at 24 weeks of age following euthanasia for downstream metabolomic profiling. Mice were euthanized by cervical dislocation, which a trained technician performed following the IACUC-approved protocol. This method was chosen because the study required immediate excision and stabilization of liver tissue for downstream metabolomic analysis. Chemical euthanasia methods such as CO_2_ or injectable anesthetics (e.g., pentobarbital) are known to rapidly alter metabolic pathways, including glycolysis, mitochondrial respiration, energy metabolites, and redox intermediates, thereby introducing artifacts into metabolomic profiles (Molhemi et al., 2025; Wei et al., 2024; Fedorov et al., 2023; Hogarth et al., 2023; Overmyer et al., 2015; Mimura and Noma, 2025). Rapid tissue harvesting without pharmacologic interference is therefore critical, as many metabolites have half-lives of seconds (Patti et al., 2012). Cervical dislocation avoided these confounding effects and enabled near-immediate tissue collection. Its use is consistent with the AVMA Guidelines for the Euthanasia of Animals, which classify cervical dislocation as “acceptable with conditions” for mice when performed by trained personnel and when required for scientific justification (AVMA, 2020) (Association, 2020).

Targeted metabolomics was performed to quantify hepatic concentrations of lactate, pyruvate, and glucose, following previously published protocols (Mazi et al., 2019). Briefly, hydrophilic interaction chromatography coupled with quadrupole time–of–flight mass spectrometry (HILIC–QTOF MS) was employed. Data acquisition was conducted in both positive and negative electrospray ionization modes. Metabolite annotation and identification were achieved by matching experimental retention times and mass–to–charge ratios against both *in silico* predictions and in–house spectral libraries using MS–DIAL software. To ensure data accuracy and reproducibility, internal standards were included in all analytical runs. Before statistical testing, metabolite distributions were examined for normality. As the data did not conform to a Gaussian distribution, the Johnson Su algorithm was applied to achieve normalization. Statistical comparisons between wild–type and tx–j mice were performed using an unpaired Student’s *t*–test, assuming equal variance (assessed using F–value and associated probability).

## Results

The level of heterogeneity across the 13 studies was considerable (I^2^ ≥ 50%; mean ± SD = 76 ± 26; Table 3; Supplementary Information), mainly observed in most liver-related studies, with one study also examining both liver and peripheral mononuclear cells (PBMCs). This substantial heterogeneity likely resulted from variations in species (humans, mice, rats, dogs), WD models (including diagnoses of WD in humans, *Atp7b* ^*tx/tx*^, *Atp7b* ^−/−^, Long–Evans Cinnamon, Long Evans Agouti Piebald, and TX–J/TX–R mouse models), ages of subjects, tissue preparation techniques, and the specific mitochondrial measures (such as copper levels, enzyme activity, and morphology). Due to these differences, random–effects models were used to better account for both within–and between–study variability, yielding more cautious and broadly applicable effect estimates.

To facilitate interpretation of the findings from these studies, we organized the reported outcomes into major functional domains of mitochondrial biology. This categorization enabled a more precise comparison across models and study designs, while also highlighting the breadth of mitochondrial disturbances observed in WD. Specifically, the 13 studies (Nagasaka et al., 2006; Gu et al., 2000; Medici et al., 2020; Mansouri et al., 1997; Sokol et al., 1994; Roberts et al., 2008; Polishchuk et al., 2019; Einer et al., 2019; Lichtmannegger et al., 2016; Zischka et al., 2011; Dong et al., 2015; Ohhira et al., 1995; Sauer et al., 2011) were grouped into six categories: mitochondrial copper content, mitochondrial morphology, oxidative stress markers, mtDNA copy number, oxygen–linked ATP production, and mitochondrial Complex activities (Table 3). To further interpret these findings, we assessed the certainty of evidence for each outcome using the GRADE framework, which considers risk of bias, inconsistency, indirectness, imprecision, and potential publication bias. Most studies were preclinical, and human studies were limited in sample size, resulting in moderate concern for bias. Heterogeneity across outcomes was substantial, and the inclusion of multiple species—including humans, mice, rats, and dogs—introduced indirectness that may limit the applicability of findings to human disease. Imprecision due to small numbers of studies and wide confidence intervals further reduced certainty, and potential publication bias could not be excluded. Overall, outcomes with large effect sizes and statistically significant results, such as mitochondrial copper accumulation, Complex IV activity, and citrate synthase activity in older or adult subjects, were rated as moderate certainty. In contrast, outcomes that were non-significant or supported by only a few studies were rated as having low certainty (Table 3). Only one study (Polishchuk et al., 2019) directly examined mitochondria–lysosome interactions and autophagy pathways in WD. Therefore, a pooled estimate and heterogeneity (I^2^) could not be calculated. This single study reported activation of autophagy in hepatic tissue from patients and *Atp7b* ^*−/−*^ models, suggesting a protective response against copper–induced mitochondrial injury. However, as a single study, its risk of bias is unclear, the inconsistency is not assessable, and the indirectness is moderate (model-based hepatic data only). Additionally, there is limited precision (no quantitative synthesis), and the publication bias is unknown. As such, the GRADE certainty is very low.

These certainty ratings provided context for interpreting the subsequent analyses of each mitochondrial functional domain, highlighting which findings are supported by more robust evidence and which should be considered preliminary or exploratory. Each mitochondrial outcome is described in detail below.

### Mitochondrial copper content

Because copper accumulation is the hallmark biochemical abnormality in WD (Zischka and Lichtmannegger, 2014; Zischka and Einer, 2018), assessing mitochondrial copper levels provides direct insight into the primary toxic insult driving mitochondrial dysfunction. Six studies—spanning humans, dogs, mice, and rats—assessed mitochondrial copper levels, all using liver biopsies (Sokol et al., 1994; Polishchuk et al., 2019; Einer et al., 2019; Lichtmannegger et al., 2016; Zischka et al., 2011; Dong et al., 2015). Subject ages varied: human participants averaged 25 years, *Atp7b* ^−/−^ mice were studied at 10 months, and rats ranged from 1 to 6 months, with one study further subdividing disease progression into affected, onset, diseased, and moribund stages (indicated by one to four asterisks in Figure 1B). Regardless of the model, all studies consistently showed elevated hepatic mitochondrial copper levels. A random–effects meta–analysis yielded a large standardized mean difference (SMD) ± standard error (SE) of 6.7 ± 0.9 (P < 0.001; Table 3), underscoring the robustness of this copper overload phenotype. Given the small number of studies (n = 6), publication bias could not be reliably assessed.

#### Mitochondrial morphology

**FIGURE 2:**
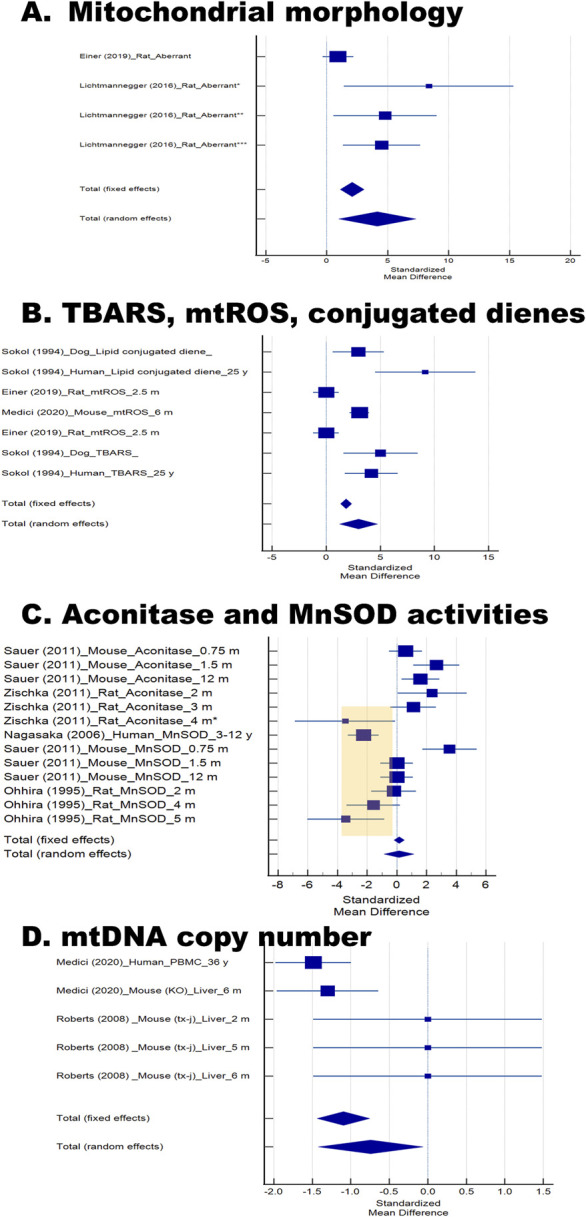
Forest plots of mitochondrial morphology, oxidative stress markers, and mtDNA copy number in Wilson disease (WD) and related models. Forest plots were generated from data extracted from the 13 studies included in the final analysis. These analyses collectively evaluate structural, oxidative, and genomic mitochondrial alterations in WD. The figure is organized into four panels: **(A)** Mitochondrial morphology, expressed as the percentage of aberrant mitochondria. In the Lichtmannegger study, one to four asterisks indicate disease stage: affected (*), onset ( ** *),* diseased (***), and moribund (****). **(B)** Markers of lipid peroxidation and membrane oxidative damage, including TBARS, mitochondrial reactive oxygen species (mtROS), and conjugated dienes. **(C)** Krebs’ cycle and antioxidant enzyme activities, specifically aconitase and manganese superoxide dismutase (MnSOD). In the Zischka study, an asterisk denotes the clinically apparent disease stage. Highlighted studies are discussed in the text. **(D)** Mitochondrial DNA (mtDNA) copy number comparison between WD and experimental models and controls. For all panels, individual studies are represented by markers showing the standardized mean difference (Hedges’ *g*), with horizontal lines denoting 95% confidence intervals (CI). Marker size reflects study weight under fixed–effects and random–effects models. Diamonds at the bottom of each panel indicate pooled estimates for both fixed–and random–effects models, with their ranges corresponding to the 95% CI. Studies are labeled by first author, year of publication, species, and (where reported) age.

Structural integrity of mitochondria is essential for maintaining energy production, and morphological abnormalities often reflect underlying biochemical stress. Two studies, both using rats aged ∼2–3 months, investigated mitochondrial ultrastructure by electron microscopy (Einer et al., 2019; Lichtmannegger et al., 2016). One of these studies stratified animals by disease stage (affected, onset, diseased; marked by one to three asterisks in Figure 2A). Pooled analysis showed a significant increase in structurally abnormal mitochondria in WD models compared with controls (SMD ±SE = 4 ± 2, P = 0.012; Table 3). These findings align with the biochemical evidence of copper–induced mitochondrial stress and provide a morphological correlate of mitochondrial dysfunction (Figure 2A).

### Oxidative stress markers

Oxidative damage is a major downstream consequence of copper toxicity, making it a critical readout for understanding the functional impact of mitochondrial injury in WD. Seven studies evaluated indices of oxidative stress (Nagasaka et al., 2006; Medici et al., 2020; Sokol et al., 1994; Einer et al., 2019; Zischka et al., 2011; Ohhira et al., 1995; Sauer et al., 2011), with outcomes stratified into markers expected to increase such as markers of mitochondrial membrane oxidative damage (e.g., thiobarbituric acid–reactive substances or TBARS, conjugated dienes; Figure 2B) and mitochondrial reactive oxygen species (mtROS; Figure 2B), and those expected to decrease (e.g., aconitase and MnSOD activities; Figure 2C).

In the first study (Einer et al., 2019 (Einer et al., 2019)), male and female *Atp7b* ^*−/−*^ rats, aged around 2.5 months, were compared with *Atp* ^*+/−*^ controls. Liver mitochondria exhibited increased H_2_O_2_ production (mtROS) when supplied with either malate–glutamate or succinate. However, in the forest-plot–type comparison across biomarkers and studies, these increases did not reach statistical significance relative to the other models or markers. These rats were likely relatively young compared to other disease models, reflecting earlier disease stages in which cumulative mitochondrial damage has not yet produced significant oxidative shifts. In the second study (Medici et al., 2020), liver mitochondria from C3He-*Atp7b* ^*tx-J*^ (“tx-j”) mice (both sexes) showed higher mtROS production via Complex III. In the third study (Sokol et al., 1994), fresh liver biopsies from human patients with Wilson disease (both sexes) and from Bedlington terriers with copper toxicosis were used; isolated hepatic mitochondria (and microsomes in humans) were studied. Lipid peroxidation (measured as conjugated dienes and TBARS) was **significantly elevated** in mitochondria from both WD patients and affected terriers.

Studies assessing an enzyme that is a target of oxidative stress damage (i.e., aconitase (Zischka et al., 2011; Sauer et al., 2011)) in livers from mice (aged 0.75–12 m) and rats (aged 2–4 m) and an enzymatic defense (MnSOD (Nagasaka et al., 2006; Ohhira et al., 1995; Sauer et al., 2011);) in liver biopsies from humans (aged 6–18 y), mice (aged 0.75–12 m), and rats (2–5 m old) yielded no overall differences between WD and controls (Figure 2C). Closer inspection, however, revealed that aconitase activity was reduced in rats at 4 months of age, and MnSOD activity was reduced in human liver biopsies and in rats aged 4–5 months, suggesting that loss of antioxidant capacity emerges with disease progression in affected individuals and older/clinically affected rodents (Figure 2C, highlighted).

#### mtDNA copy number

Because mitochondria harbor their own genome, mtDNA content serves as an indicator of mitochondrial biogenesis and integrity, both of which may be disrupted in WD. Two studies examined mtDNA content, one using human PBMCs (Medici et al., 2020) and the other rodent liver tissue (Roberts et al., 2008). Decreased mtDNA copy number was observed in PBMCs from adult humans and liver mitochondria from 6–months–old *Atp7b* ^−/−^ mice, whereas TX–J mice aged 2–6 months showed no change. Pooled analysis indicated a modest but significant reduction overall (SMD ±SE = −0.7 ± 0.3, P = 0.032; Figure 2D; Table 3), consistent with impaired mitochondrial genome maintenance in WD.

#### Oxygen–linked ATP production

**FIGURE 3:**
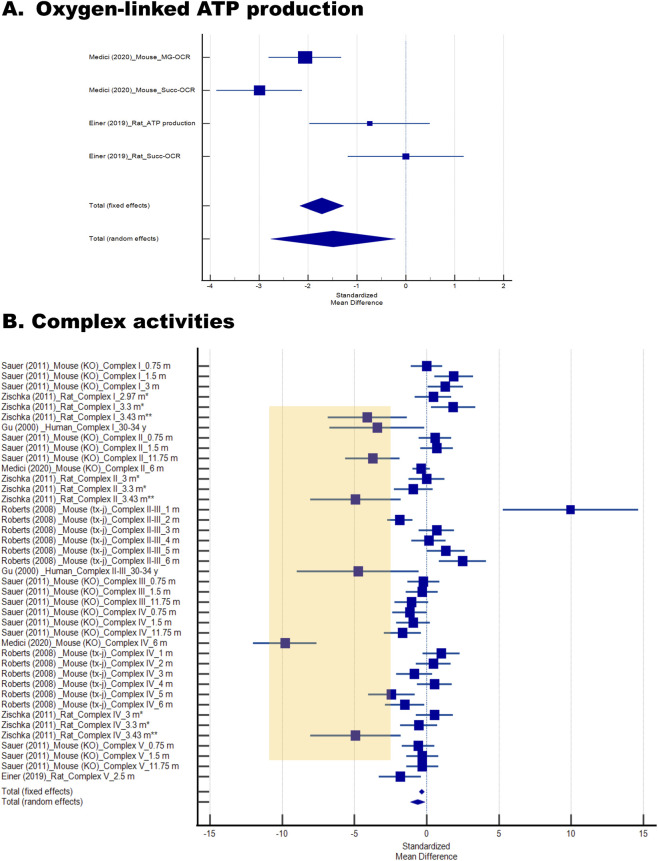
Forest plots of oxygen-linked ATP production, and respiratory chain Complex activities in Wilson disease (WD) and related models. Forest plots were generated to evaluate mitochondrial bioenergetics across included studies. **(A)** Oxygen–linked ATP production measured using either malate–glutamate (MG) or succinate (Succ) as respiratory substrates. **(B)** Respiratory chain Complex activities, reported for individual Complexes (I–IV). Studies are labeled by first author, year of publication, species, Complex number (in Roman numerals), and age of subjects. In the Zischka study, one asterisk denotes a preclinical stage, while two asterisks indicate clinically apparent disease. Values highlighted in orange indicate the lowest activities, which were generally observed in studies of older or older, affected subjects.

ATP synthesis represents the central bioenergetic function of mitochondria, and measuring oxygen–linked ATP production provides a direct assessment of mitochondrial performance. Two studies using *Atp7b* ^−/−^ mice (ages 2.2–2.5 months and 6 months) assessed oxygen–linked ATP synthesis with succinate or malate–glutamate substrates (Medici et al., 2020; Einer et al., 2019). Both reported impaired capacity for ATP production, with meta–analysis confirming a significant reduction (SMD ±SE = −1.5 ± 0.6, P = 0.023; Figure 3A; Table 3). This defect highlights the functional consequences of mitochondrial injury beyond structural abnormalities and copper overload.

### Mitochondrial Complex activities

**FIGURE 4:**
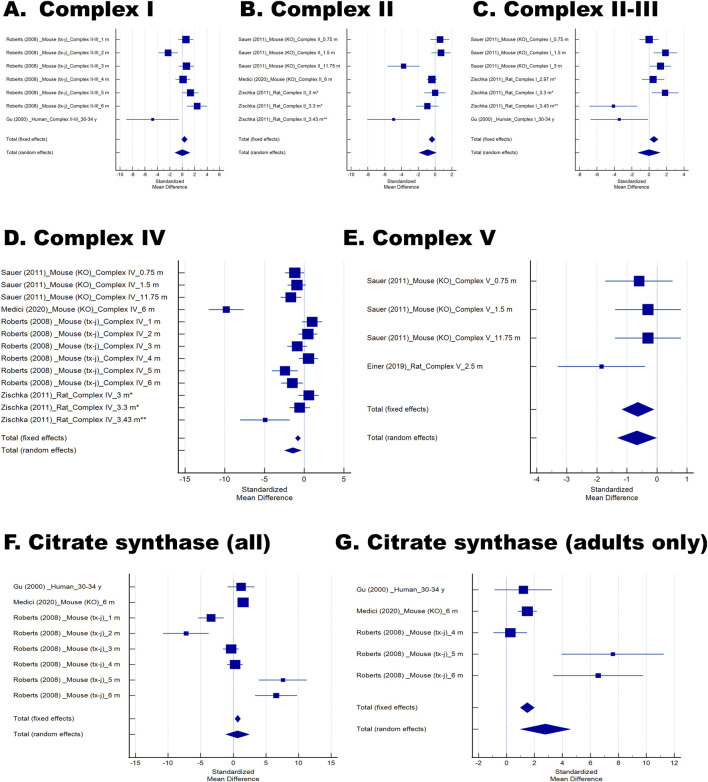
Forest plots of Complex I–V and citrate synthase activities in Wilson disease (WD) and related models. Forest plots of Complex (I) **(A)**, II **(B)**, II–III **(C)**, IV **(D)**, and V **(E)** activities **(F)**. Citrate synthase activity across all studies included in the analysis. **(G)** Citrate synthase activity restricted to adult subjects to assess age–related effects. In all panels, individual studies are represented by markers showing the standardized mean difference (Hedges’ *g*), with horizontal lines denoting 95% confidence intervals (CI). Marker size reflects the relative study weight under fixed–effects and random–effects models. Pooled estimates for both models are displayed as diamonds at the bottom of each plot, with diamond width corresponding to the 95% CI.

Activities of the electron transport chain Complexes (I–V) reflect the efficiency of oxidative phosphorylation and are highly sensitive to copper-mediated damage. Six studies (Gu et al., 2000; Medici et al., 2020; Roberts et al., 2008; Einer et al., 2019; Zischka et al., 2011; Sauer et al., 2011) evaluated respiratory chain Complex activities in liver biopsies from humans (ages 30–34), *Atp7b* ^−/−^ mice (0.75–12 months), TX–J mice (1–6 months), and rats (3–3.5 months, both symptomatic and asymptomatic; Figure 3B). Across species, activities of Complexes I–V were reduced, with a pooled SMD ±SE of −0.6 ± 0.3 (P = 0.013; Table 3). Declines were most pronounced in older animals and clinically affected subjects, paralleling findings in human samples (Figure 3B, highlighted in orange). When analyzing the activity of each Complex individually (Figures 4A–E), only Complex IV and V showed statistically significant decreases (−1.4 ± 0.5 P = 0.008 and −0.7 ± 0.3 P = 0.044; Figures 4D,E), with Complex IV activity being the most affected (Table 3). It is interesting to note that Complex IV activity was lower in KO mice from 0.75 m to 12 m; however, in TX–J, the most pronounced changes were observed in older animals (5–6 m old) and in rats when they were affected clinically (3.43 m old).

Interestingly, three studies also reported citrate synthase activity as a marker of mitochondrial content (Gu et al., 2000; Medici et al., 2020; Roberts et al., 2008). While no overall difference was seen (SMD ±SE = 0.7 ± 0.9, P = 0.481; Figure 4F; Table 3), subgroup analysis of adult subjects (humans and rodents ≥4 months old) revealed significantly increased activity (SMD ±SE = 2.8 ± 0.9, P = 0.003; Figure 4G; Table 3), potentially reflecting compensatory mitochondrial proliferation or biogenesis.

Taken together, these analyses reveal a coherent picture of mitochondrial dysfunction in WD, characterized by pronounced hepatic mitochondrial copper accumulation, increased oxidative stress, impaired ATP production, and reduced activity of respiratory Complex IV, while compensatory increases in citrate synthase activity suggest attempts at mitochondrial adaptation (GRADE certainty moderate; Table 3). Structural derangements, and loss of mtDNA integrity accompanied these changes. Although the limited number of studies and heterogeneity necessitate cautious interpretation, the convergence of evidence across species and models underscores mitochondrial impairment as a central pathogenic feature of WD.

Since a key unresolved issue is whether the decline in OXPHOS is accompanied by increased glycolysis to sustain energy demands, and given the limited literature addressing this aspect, we analyzed hepatic levels of glucose, pyruvate, and lactate using metabolomics in KO and control 6–months–old mice. Lactate, pyruvate, and glucose were selected for analysis because they represent key nodes at the intersection of mitochondrial respiration and glycolysis. Pyruvate is the end product of glycolysis and a primary substrate for the tricarboxylic acid cycle within mitochondria. Its relative partitioning toward lactate reflects the balance between mitochondrial oxidative metabolism and anaerobic glycolysis. Glucose, as the upstream substrate of glycolysis, provides a broader context for interpreting these metabolic shifts. Our results showed that, consistent with a deficit in OXPHOS, the lactate–to–pyruvate ratio was higher in KO mice compared to controls (mean ± SD for KO and WT: 0.5 ± 0.9 and −1.6 ± 0.8; *t* (Fedorov et al., 2023) = −4.0, P = 0.0003, Cohen’s *d* = 1.29) and the pyruvate–to–glucose ratio was lower (−0.4 ± 0.9 and 0.4 ± 0.9; *t* (Fedorov et al., 2023) = 3.0, P = 0.004, Cohen’s *d* = 0.89). However, there was no evidence of increased glycolytic flux, as the ratios of lactate–to–glucose were not statistically different between the groups (−0.8 ± 0.9 and −0.7 ± 0.7; *t* (Fedorov et al., 2023) = 0.50, P = 0.617, Cohen’s *d* = 0.12).

## Discussion

This meta–analysis demonstrates that hepatic mitochondrial dysfunction is a central feature of WD across humans and animal models. Mitochondria consistently accumulate copper, and display impaired oxygen–dependent ATP synthesis, confirming them as primary targets of copper toxicity (Table 3). The substantial heterogeneity observed across studies (mean I^2^ ≈ 76%) reflected the wide diversity of experimental designs, species, and analytical methods used to investigate mitochondrial alterations in WD and related models. Differences in genetic backgrounds (e.g., *Atp7b* ^*−/−*^, LEC, and other rodent models), disease stage, tissue type, age, and biochemical or imaging endpoints likely contributed to variable effect sizes. While such variability underscores the biological and methodological complexity of WD, it also limits the precision of pooled estimates and should temper overinterpretation of the aggregated effect magnitudes. The use of random–effects models was therefore appropriate, as it accommodated both within–and between–study variability and yielded more conservative summary estimates. Importantly, this heterogeneity underscores the need for standardized outcome definitions—such as consistent measures of mitochondrial respiration, copper content, and oxidative stress markers—to facilitate more reliable cross-study comparisons and enhance future meta–analytic inference.

Oxidative stress emerged as a major contributor to mitochondrial damage. Markers such as TBARS and mitochondrial ROS were elevated, while oxidative damage targets or antioxidant defenses (aconitase, MnSOD) declined with disease progression in affected individuals and older/clinically affected rodents. This pattern suggests that defenses are initially intact but deteriorate over time, amplifying oxidative injury. Similarly, Complex activities declined more severely in older or symptomatic subjects, while increased citrate synthase activity indicated compensatory biogenesis. Reduced mtDNA copy number underscores the mitochondrial genome’s vulnerability to copper–induced oxidative stress.

Model differences partly explain heterogeneity. *Atp7b* ^−/−^ mice show a consistent liver phenotype but lack genetic diversity, while toxic milk and TX substrains resemble human missense mutations yet differ in onset. LEC and LPP rats develop fulminant hepatitis with no evidence of neurological symptoms, and Bedlington terriers highlight genetic complexity (Table 1). Despite these differences, the mitochondrial defects across models closely mirror human findings, strengthening their translational value. Notably, several abnormalities—including copper buildup, oxidative stress, and mtDNA loss—appear even in presymptomatic WD–affected humans, suggesting earlier mitochondrial vulnerability compared with animals. Differences in lifespan, mutation spectrum, and antioxidant capacity may explain this discrepancy, underscoring the need for early detection in patients.

The combined findings from our analysis suggest a link between mitochondrial genome instability and functional decline. While under high oxidative stress conditions, the mtDNA copy number *per* cell and mtDNA deletions are usually increased (Giulivi et al., 2010) in an attempt to maintain a normal level of “undamaged” template without increases in either OXPHOS or mitochondrial mass (Giulivi et al., 2010; Lee et al., 1998), the concordant reduction in mtDNA copy number and the selective loss of Complex IV and V activities suggest a mtDNA depletion–like mechanism in WD (Medici et al., 2020), compounded by direct copper-driven impairment of Complex IV assembly/metallation (Timon-Gomez et al., 2018; Ruiz et al., 2021; Nyvltova et al., 2022). Increased citrate synthase activity suggests expanded mitochondrial mass but reduced effective OXPHOS (ratio of Complex IV to citrate synthase), echoing features of mtDNA depletion syndromes (Moraes et al., 1991; Oskoui et al., 2006).

We examined whether impaired OXPHOS was offset by enhanced glycolysis. In *Atp7b* ^−/−^ mice, lactate–to–pyruvate ratios were elevated and pyruvate–to–glucose ratios decreased, consistent with OXPHOS deficiency (Giulivi et al., 2010). However, lactate–to–glucose ratios were unchanged, arguing against a true glycolytic shift. Prior studies report inconsistent glycolytic responses (Juan et al., 2005; Kraft et al., 1999), highlighting that WD pathogenesis cannot be reduced to simple copper toxicosis (Xu et al., 2015; Lai and Blass, 1984; Scheiber et al., 2011) and may vary by tissue or disease stage.

These findings have important implications. Mitochondrial copper overload likely drives hepatocellular injury (Gerosa et al., 2019; Nagasaka et al., 2006; Gu et al., 2000; Zischka and Lichtmannegger, 2014; Medici et al., 2020; Mansouri et al., 1997; Zischka and Einer, 2018; Sokol et al., 1994; Roberts et al., 2008; Klein et al., 1998; Polishchuk et al., 2019; Sternlieb, 1992; Einer et al., 2019; Lichtmannegger et al., 2016; Zischka et al., 2011; Li et al., 1991; Dong et al., 2015; Ohhira et al., 1995; Sauer et al., 2011; Einer et al., 2023; Kim et al., 2021; Lee et al., 2011; Sternlieb, 1968; Sternlieb and Feldmann, 1976; Sternlieb et al., 1995; Szeligowska et al., 2022; Yamamoto et al., 2001; Yurkova et al., 2011), while age–related declines in antioxidant defenses and Complex activities emphasize the need for early therapeutic intervention. Compensatory responses, such as increased citrate synthase activity, could represent therapeutic targets. Animal models remain invaluable for mechanistic studies and drug development, though species–and model–specific differences must be considered when extrapolating to humans.

Mitochondrial injury should also be considered within the broader cellular context. Lysosomes act as early copper storage sites (Klein et al., 1998; Terada et al., 1999), but their dysfunction may impair mitophagy and removal of damaged mitochondria. This dual disturbance promotes copper accumulation, oxidative stress, and persistent mitochondrial injury. Importantly, several abnormalities are detectable in asymptomatic patients, suggesting that mitochondrial dysfunction arises early and contributes to disease onset. Only one eligible study (Polishchuk et al., 2019) directly examined mitochondria–lysosome interactions in WD. That study reported activation of autophagy in ATP7B-deficient hepatocytes and human liver tissue, which protected cells from copper-induced mitochondrial damage and apoptosis. These findings suggest that stimulating autophagy or enhancing lysosome–mitochondria communication might represent a compensatory, rather than defective, mechanism in WD. Given that quantitative synthesis was not possible, the certainty of this evidence was rated as very low (GRADE), and further studies are needed to determine whether autophagy activation is consistently protective across models and disease stages.

Mitochondrial copper overload and the resulting impairment of the respiratory chain point to several translational avenues that could be explored in WD. First, stimulating mitochondrial biogenesis is an attractive strategy for restoring respiratory capacity and replacing dysfunctional mitochondria. Pharmacologic activators of the PGC–1α pathway (for example, bezafibrate, resveratrol, and other small molecules shown to increase PGC–1α activity) increase mitochondrial mass and OXPHOS capacity in preclinical models and have been proposed as therapeutic adjuncts in disorders of mitochondrial dysfunction (Abu Shelbayeh et al., 2023; Hofer et al., 2014). Such agents could be tested in WD models to determine whether boosting biogenesis ameliorates copper-induced respiration defects.

Second, targeted antioxidant support may blunt the downstream oxidative damage caused by copper–driven ROS generation. Mitochondria–directed antioxidants (MnSOD mimetics) and classic mitochondrial antioxidants (coenzyme Q10 or CoQ10, α-lipoic acid) have a substantial preclinical and some clinical track record in limiting mitochondrial oxidative injury and improving bioenergetics in mitochondrial disease contexts; these could be repurposed or trialed as adjuncts in WD to reduce ROS–mediated respiratory chain damage while chelation therapies lower copper load. Clinical experience with CoQ10 in mitochondrial cytopathies provides precedent for safety and measurable biochemical endpoints (Mantle et al., 2024; Glover et al., 2010; Grujicic and Allen, 2024).

Third, our findings strengthen the rationale for *mitochondria*–*targeted copper chelation*. Peptides such as methanobactin—which bind copper with high affinity and have shown efficacy in animal models of WD with preferential mitochondrial copper clearance—represent a conceptually attractive complement to systemic chelators because they can directly address the organelle most affected by copper toxicity. Preclinical data indicate that methanobactin–type agents facilitate the rapid removal of copper from the liver and mitochondria, thereby restoring mitochondrial function (Lichtmannegger et al., 2016; Einer et al., 2023). However, the human translation of these findings remains to be established (Kaler, 2016).

Finally, enhancing mitochondrial quality control could mitigate the accumulation of dysfunctional, copper-loaded mitochondria. Interventions that upregulate mitophagy (PINK1/Parkin pathway modulators, or agents that improve lysosome–mitochondria crosstalk) may promote selective removal of damaged mitochondria and restore cellular homeostasis. There is a growing mechanistic literature linking PINK1/Parkin–mediated mitophagy and lysosomal–mitochondrial biogenesis crosstalk (Jin and Youle, 2012; Liu et al., 2023). Targeted manipulation of these pathways in WD models may reveal whether augmenting mitochondrial turnover improves bioenergetics and reduces cell injury.

We note important caveats: (*i*) many candidate approaches remain at preclinical stages and their effect on systemic copper balance and on neurological vs. hepatic phenotypes may differ; (*ii*) mitochondrial biogenesis without effective removal of copper may exacerbate copper incorporation into newly formed mitochondria, so combined strategies (for example, simultaneous chelation *and* biogenesis or antioxidants) may be required; and (*iii*) appropriate biomarkers (mitochondrial respiration assays, organellar copper measurements, oxidative stress markers) and validated WD animal models will be essential to prioritize candidates for clinical testing.

Limitations of this meta-analysis include the relatively small number of available studies and substantial heterogeneity in reported outcomes, methodologies, and model systems. Most datasets focused on hepatic tissue, which may not fully represent extrahepatic or neurological copper toxicity. In addition, treated cohorts were underrepresented—most investigations examined untreated or early–stage WD, leaving uncertainty about the extent and reversibility of mitochondrial dysfunction after chelation or zinc therapy. Temporal information was also limited, as few studies provided longitudinal data to track mitochondrial alterations across disease progression. Mechanistic integration remains incomplete, as most reports rely on biochemical or ultrastructural analyses without parallel transcriptomic, proteomic, or metabolomic profiling to link molecular pathways with functional outcomes. Finally, species and model variability (e.g., *Atp7b* ^*−/−*^, LEC, LPP, and other rodent or cellular systems) introduces biological heterogeneity that may affect generalizability to human disease. Nevertheless, convergence of findings across species and methodological approaches strongly supports a central role of mitochondrial dysfunction in WD pathogenesis.

In conclusion, WD is characterized by mitochondrial copper accumulation, structural damage, impaired bioenergetics, oxidative stress, and mtDNA loss. These abnormalities emerge early and worsen with disease progression, likely exacerbated by defective lysosomal copper handling and impaired mitophagy. Future work should expand to extrahepatic tissues, clarify longitudinal changes, and explore therapeutic strategies aimed at preserving mitochondrial integrity and inter–organelle communication.

### Lay summary

In Wilson disease, a genetic disorder of copper metabolism, excess copper accumulates in the liver cell mitochondria—the cell’s energy factories—where it causes structural damage and disrupts energy production. These changes appear early in the disease and worsen over time, leading to higher oxidative stress and loss of mitochondrial DNA. Together, this shows that mitochondrial injury is a key driver of Wilson disease and could be an essential target for future treatments.

## Data Availability

The original contributions presented in the study are included in the article/Supplementary Material, further inquiries can be directed to the corresponding author.
