# Supplementary material for: Mitochondrial dysfunction in Wilson disease: a systematic review and meta–analysis across human and animal models
Source: Front Mol Biosci. 2025 Dec 17;12:1712573. doi: 10.3389/fmolb.2025.1712573 (PMC12753413; doi:10.3389/fmolb.2025.1712573)
Supplement: Supplementary file 1 [file DataSheet1.pdf]

PRISMA 2020 Checklist

Section/Topic	Item #	Checklist Item	Location in Manuscript
**Title**	1	Identify the report as a systematic review, meta-analysis, or both.	Title page
**Abstract**	2	Structured summary including objectives, eligibility criteria, information sources, risk of bias, results, conclusions.	Abstract
**Introduction**	3	Describe the rationale for the review in the context of existing knowledge.	Introduction
	4	Provide an explicit statement of objectives or questions.	Introduction
**Methods**	5	Specify inclusion and exclusion criteria for study selection.	Methods
	6	Specify information sources (databases, registers, websites, organizations) with dates of last search.	Methods
	7	Present full search strategies for all sources, including filters and limits.	Methods
	8	Explain the process for selecting studies (screening, eligibility, inclusion).	Methods; PRISMA flow diagram
	9	Describe the process for data extraction and items extracted.	Methods
	10	Describe any methods for risk of bias assessment in included studies.	Methods
	11	Specify effect measures used for each outcome.	Methods
	12	Describe methods for synthesizing results, handling heterogeneity, and sensitivity analyses.	Methods
	13	Describe reporting bias assessment.	Methods
	14	Describe certainty or confidence assessment (e.g., GRADE).	Methods
**Results**	15	Give number of studies screened, assessed, included; provide reasons for exclusions at each stage.	Methods; Results; PRISMA flow diagram
	16	Cite each included study and summarize key characteristics.	Results
	17	Present risk of bias assessments.	Results; Tables 1–2
	18	For all outcomes, present summary estimates, effect sizes, confidence intervals, and heterogeneity.	Results; Tables 2–3
	19	Present results of individual studies and syntheses (e.g., forest plots).	Results; Figures 1–3
	20	Present results of sensitivity or subgroup analyses.	Results
	21	Present assessments of reporting bias.	Results
	22	Present certainty of evidence for each outcome.	Results
**Discussion**	23	Interpret results in context of other evidence.	Discussion
	24	Discuss limitations of evidence included in the review.	Discussion
	25	Discuss limitations of the review process itself.	Discussion
	26	Discuss implications for practice, policy, and future research.	Discussion
**Other Information**	27	Report sources of financial support and conflicts of interest.	End of manuscript / Funding & COI statements

Search Strategy for each database

PubMed

Search: (Wilson disease OR Wilson's disease OR copper toxicity) AND (mitochondrial OR mitochondria OR mitochondrion OR oxidative stress OR free radic*) AND (english[Filter])

(("hepatolenticular degeneration"[MeSH Terms] OR ("hepatolenticular"[All Fields] AND "degeneration"[All Fields]) OR "hepatolenticular degeneration"[All Fields] OR ("wilson"[All Fields] AND "disease"[All Fields]) OR "wilson disease"[All Fields] OR ("hepatolenticular degeneration"[MeSH Terms] OR ("hepatolenticular"[All Fields] AND "degeneration"[All Fields]) OR "hepatolenticular degeneration"[All Fields] OR ("wilson s"[All Fields] AND "disease"[All Fields]) OR "wilson s disease"[All Fields]) OR (("copper"[Supplementary Concept] OR "copper"[All Fields] OR "copper"[MeSH Terms] OR "coppers"[All Fields] OR "copper s"[All Fields]) AND ("toxic"[All Fields] OR "toxical"[All Fields] OR "toxically"[All Fields] OR "toxicant"[All Fields] OR "toxicant s"[All Fields] OR "toxicants"[All Fields] OR "toxicated"[All Fields] OR "toxication"[All Fields] OR "toxicities"[All Fields] OR "toxicity"[MeSH Subheading] OR "toxicity"[All Fields] OR "toxicity s"[All Fields] OR "toxics"[All Fields]))) AND ("mitochondria"[MeSH Terms] OR "mitochondria"[All Fields] OR "mitochondrial"[All Fields] OR "mitochondrially"[All Fields] OR "mitochondrials"[All Fields] OR ("mitochondria"[MeSH Terms] OR "mitochondria"[All Fields] OR "mitochondria s"[All Fields] OR "mitochondriae"[All Fields] OR "mitochondrias"[All Fields]) OR ("mitochondria"[MeSH Terms] OR "mitochondria"[All Fields] OR "mitochondrion"[All Fields] OR "mitochondrion s"[All Fields] OR "mitochondrions"[All Fields]) OR ("oxidative stress"[MeSH Terms] OR ("oxidative"[All Fields] AND "stress"[All Fields]) OR "oxidative stress"[All Fields]) OR ("free"[All Fields] AND "radic*"[All Fields])) AND "english"[Language])

N = 7926

Scopus

("mitoch*" OR "oxidative stress" OR "free radic*") AND ("Wilson's disease" OR "Wilson disease" OR "hepatolenticular degeneration" OR "copper toxicity")

N = 2315

SciFinder

("mitoch*" OR "oxidative stress" OR "free radic*") AND ("Wilson's disease" OR "Wilson disease" OR "hepatolenticular degeneration" OR "copper toxicity")

N = 1432

MITOCHONDRIAL COPPER

Meta-analysis: continuous measure

Variable for studies	Studies
1. Intervention groups	
Variable for number of cases	n_mut<br>n-mut
Variable for mean	mean_mut<br>mean-mut
Variable for SD	SD_mut<br>SD-mut
2. Control groups	
Variable for number of cases	n
Variable for mean	mean
Variable for SD	SD

Study	N1	N2	Total	SMD	SE	95% CI	t	P	Weight (%)	
									Fixed	Random
Sokol (1994)_Dog	4	4	8	2.425	0.863	0.313 to 4.537			5.45	7.08
Sokol (1994)_Human	3	8	11	5.950	1.411	2.757 to 9.142			2.04	6.32
Dong ((2015)_Mouse	10	12	22	1.557	0.474	0.568 to 2.545			18.08	7.46
Einer (2019)_Rat	6	6	12	3.502	0.891	1.515 to 5.488			5.11	7.05
Lichtmannegger (2016)_Rat*	4	15	19	10.488	1.784	6.724 to 14.253			1.28	5.73
Lichtmannegger (2016)_Rat*	11	9	20	2.148	0.548	0.996 to 3.300			13.51	7.40
Lichtmannegger (2016)_Rat**	4	15	19	39.682	6.460	26.053 to 53.311			0.097	1.43
Lichtmannegger (2016)_Rat**	6	9	15	4.059	0.892	2.133 to 5.986			5.11	7.05
Lichtmannegger (2016)_Rat***	4	9	13	5.855	1.277	3.044 to 8.665			2.49	6.53
Lichtmannegger (2016)_Rat****	3	15	18	12.266	2.131	7.748 to 16.784			0.89	5.19
Polishchuk (2019)_Rat*	24	37	61	6.155	0.614	4.925 to 7.384			10.76	7.34
Polishchuk (2019)_Rat***	21	37	58	2.938	0.383	2.170 to 3.706			27.63	7.52
Zischka (2011)_Rat	2	11	13	4.650	1.159	2.100 to 7.200			3.03	6.70
Zischka (2011)_Rat	13	11	24	8.919	1.347	6.126 to 11.712			2.24	6.42
Zischka (2011)_Rat	6	11	17	14.666	2.561	9.207 to 20.124			0.62	4.54
Zischka (2011)_Rat	17	11	28	11.419	1.572	8.189 to 14.649			1.65	6.07
Zischka (2011)_Rat	11	11	22	134.456	20.274	92.165 to 176.748			0.0099	0.17
Total (fixed effects)	149	231	380	3.747	0.202	3.350 to 4.143	18.587	<0.001	100.00	100.00
Total (random effects)	149	231	380	6.694	0.857	5.009 to 8.380	7.809	<0.001	100.00	100.00

Test for heterogeneity

Q	217.4958
DF	16
Significance level	$P < 0.0001$
I^2 (inconsistency)	92.64%
95% CI for I^2	89.72 to 94.74

Publication bias

Egger's test	
Intercept	5.5227
95% CI	3.7737 to 7.2716
Significance level	$P < 0.0001$
Begg's test	
Kendall's Tau	0.7500
Significance level	$P < 0.0001$

OXYGEN-LINKED ATP PRODUCTION

Meta-analysis: continuous measure

Variable for studies	Studies
1. Intervention groups	
Variable for number of cases	n_Atp7b_<br>n_Atp7b-/-
Variable for mean	mean__Atp7b_<br>mean _Atp7b-/-
Variable for SD	SD_Atp7b_<br>SD_Atp7b-/-
2. Control groups	
Variable for number of cases	n
Variable for mean	mean
Variable for SD	SD

Study	N1	N2	Total	SMD	SE	95% CI	t	P	Weight (%)	
									Fixed	Random
Medici (2020)_Mouse_MG-OCR	22	22	44	-2.063	0.369	-2.807 to -1.319			37.87	26.44
Medici (2020)_Mouse_Succ-OCR	22	22	44	-2.996	0.436	-3.875 to -2.117			27.16	25.56
Einer (2019)_Rat_ATP production	6	6	12	-0.738	0.554	-1.972 to 0.495			16.81	23.84
Einer (2019)_Rat_Succ-OCR	6	6	12	0.000	0.533	-1.187 to 1.187			18.15	24.16
Total (fixed effects)	56	56	112	-1.719	0.227	-2.169 to -1.269	-7.575	<0.001	100.00	100.00
Total (random effects)	56	56	112	-1.487	0.643	-2.762 to -0.212	-2.312	0.023	100.00	100.00

Test for heterogeneity

Q	23.0179
DF	3
Significance level	P < 0.0001
I^2 (inconsistency)	86.97%
95% CI for I^2	68.64 to 94.58

Publication bias

Egger's test	
Intercept	10.4566
95% CI	-20.3595 to 41.2727
Significance level	P = 0.2817
Begg's test	
Kendall's Tau	0.3333
Significance level	P = 0.4969

COMPLEX ACTIVITIES

Meta-analysis: continuous measure

Variable for studies	Studies
1. Intervention groups	
Variable for number of cases	n_mut<br>n-mut
Variable for mean	mean_mut<br>mean-mut
Variable for SD	SD_mut<br>SD-mut
2. Control groups	
Variable for number of cases	n
Variable for mean	mean
Variable for SD	SD

Study	N1	N2	Total	SMD	SE	95% CI	t	P	Weight (%)	
									Fixed	Random
Sauer (2011)_Mouse (KO)_Complex I_0.75 m	7	7	14	0.000	0.500	-1.090 to 1.090			3.30	2.69
Sauer (2011)_Mouse (KO)_Complex I_1.5 m	7	7	14	1.872	0.613	0.537 to 3.207			2.20	2.55
Sauer (2011)_Mouse (KO)_Complex I_3 m	7	7	14	1.298	0.557	0.0839 to 2.512			2.66	2.62
Zischka (2011)_Rat_Complex I_2.97 m*	5	6	11	0.457	0.562	-0.814 to 1.728			2.61	2.62
Zischka (2011)_Rat_Complex I_3.3 m*	5	6	11	1.832	0.677	0.300 to 3.364			1.80	2.47
Zischka (2011)_Rat_Complex I_3.43 m**	3	6	9	-4.108	1.154	-6.838 to -1.379			0.62	1.85
Gu (2000) _Human_Complex I_30-34 y	3	3	6	-3.431	1.185	-6.722 to -0.140			0.59	1.81
Sauer (2011)_Mouse (KO)_Complex II_0.75 m	7	7	14	0.592	0.513	-0.525 to 1.709			3.14	2.67
Sauer (2011)_Mouse (KO)_Complex II_1.5 m	7	7	14	0.710	0.518	-0.418 to 1.839			3.07	2.67
Sauer (2011)_Mouse (KO)_Complex II_11.75 m	7	7	14	-3.744	0.867	-5.632 to -1.856			1.10	2.22
Medici (2020)_Mouse (KO)_Complex II_6 m	22	22	44	-0.385	0.299	-0.988 to 0.218			9.23	2.88
Zischka (2011)_Rat_Complex II_3 m*	5	6	11	0.000	0.553	-1.252 to 1.252			2.69	2.63
Zischka (2011)_Rat_Complex II_3.3 m*	5	6	11	-0.914	0.587	-2.241 to 0.413			2.40	2.59
Zischka (2011)_Rat_Complex II_3.43 m**	3	6	9	-4.931	1.321	-8.054 to -1.807			0.47	1.65
Roberts (2008) _Mouse (tx-j)_Complex II-III_1 m	6	6	12	9.954	2.101	5.274 to 14.634			0.19	0.98
Roberts (2008) _Mouse (tx-j)_Complex II-III_2 m	6	97	103	-1.848	0.437	-2.714 to -0.982			4.32	2.76
Roberts (2008) _Mouse (tx-j)_Complex II-III_3 m	6	6	12	0.690	0.551	-0.538 to 1.918			2.72	2.63
Roberts (2008) _Mouse (tx-j)_Complex II-III_4 m	6	6	12	0.137	0.533	-1.052 to 1.326			2.90	2.65
Roberts (2008) _Mouse (tx-j)_Complex II-III_5 m	6	6	12	1.320	0.597	-0.0104 to 2.650			2.31	2.57
Roberts (2008) _Mouse (tx-j)_Complex II-III_6 m	6	6	12	2.472	0.734	0.837 to 4.106			1.53	2.40
Gu (2000) _Human_Complex II-III_30-34 y	3	3	6	-4.747	1.517	-8.960 to -0.534			0.36	1.44
Sauer (2011)_Mouse (KO)_Complex III_0.75 m	7	7	14	-0.218	0.502	-1.311 to 0.876			3.27	2.69
Sauer (2011)_Mouse (KO)_Complex III_1.5 m	7	7	14	-0.321	0.504	-1.419 to 0.777			3.25	2.68
Sauer (2011)_Mouse (KO)_Complex III_11.75 m	7	7	14	-1.038	0.537	-2.209 to 0.133			2.86	2.64
Sauer (2011)_Mouse (KO)_Complex IV_0.75 m	7	7	14	-1.184	0.548	-2.378 to 0.0102			2.75	2.63
Sauer (2011)_Mouse (KO)_Complex IV_1.5 m	7	7	14	-0.936	0.531	-2.092 to 0.220			2.93	2.65
Sauer (2011)_Mouse (KO)_Complex IV_11.75 m	7	7	14	-1.674	0.592	-2.964 to -0.385			2.35	2.58
Medici (2020)_Mouse (KO)_Complex IV_6 m	22	22	44	-9.800	1.086	-11.991 to -7.609			0.70	1.93
Roberts (2008) _Mouse (tx-j)_Complex IV_1 m	6	6	12	1.014	0.572	-0.259 to 2.288			2.53	2.60
Roberts (2008) _Mouse (tx-j)_Complex IV_2 m	6	6	12	0.471	0.541	-0.736 to 1.677			2.82	2.64
Roberts (2008) _Mouse (tx-j)_Complex IV_3 m	6	6	12	-0.866	0.561	-2.117 to 0.384			2.62	2.62
Roberts (2008) _Mouse (tx-j)_Complex IV_4 m	6	6	12	0.550	0.544	-0.663 to 1.763			2.78	2.64
Roberts (2008) _Mouse (tx-j)_Complex IV_5 m	6	6	12	-2.432	0.728	-4.054 to -0.809			1.56	2.40
Roberts (2008) _Mouse (tx-j)_Complex IV_6 m	6	6	12	-1.511	0.616	-2.883 to -0.140			2.18	2.55
Zischka (2011)_Rat_Complex IV_3 m*	5	6	11	0.560	0.566	-0.721 to 1.840			2.57	2.61
Zischka (2011)_Rat_Complex IV_3.3 m*	5	6	11	-0.560	0.566	-1.840 to 0.721			2.57	2.61
Zischka (2011)_Rat_Complex IV_3.43 m**	3	6	9	-4.931	1.321	-8.054 to -1.807			0.47	1.65
Sauer (2011)_Mouse (KO)_Complex V_0.75 m	7	7	14	-0.592	0.513	-1.709 to 0.525			3.14	2.67
Sauer (2011)_Mouse (KO)_Complex V_1.5 m	7	7	14	-0.296	0.503	-1.393 to 0.801			3.26	2.68
Sauer (2011)_Mouse (KO)_Complex V_11.75 m	7	7	14	-0.296	0.503	-1.393 to 0.801			3.26	2.68
Einer (2019)_Rat_Complex V_2.5 m	6	6	12	-1.845	0.652	-3.299 to -0.392			1.94	2.50
Total (fixed effects)	272	378	650	-0.345	0.0908	-0.523 to -0.167	-3.799	<0.001	100.00	100.00
Total (random effects)	272	378	650	-0.627	0.253	-1.123 to -0.130	-2.479	0.013	100.00	100.00

Test for heterogeneity

Q	289.2238
DF	40
Significance level	P < 0.0001

I^2 (inconsistency)	86.17%
95% CI for I^2	82.14 to 89.29

Publication bias

Egger's test	
Intercept	-2.6442
95% CI	-5.4071 to 0.1187
Significance level	P = 0.0602
Begg's test	
Kendall's Tau	-0.1444
Significance level	P = 0.1834

COMPLEX I ACTIVITY

Meta-analysis: continuous measure

Variable for studies	Studies
1. Intervention groups	
Variable for number of cases	n_mut
Variable for mean	mean_mut
Variable for SD	SD_mut
2. Control groups	
Variable for number of cases	n
Variable for mean	mean
Variable for SD	SD

Study	N1	N2	Total	SMD	SE	95% CI	t	P	Weight (%)	
									Fixed	Random
Sauer (2011)_Mouse (KO)_Complex I_0.75 m	7	7	14	0.000	0.500	-1.090 to 1.090			23.94	16.02
Sauer (2011)_Mouse (KO)_Complex I_1.5 m	7	7	14	1.872	0.613	0.537 to 3.207			15.96	15.30
Sauer (2011)_Mouse (KO)_Complex I_3 m	7	7	14	1.298	0.557	0.0839 to 2.512			19.30	15.67
Zischka (2011)_Rat_Complex I_2.97 m*	5	6	11	0.457	0.562	-0.814 to 1.728			18.98	15.64
Zischka (2011)_Rat_Complex I_3.3 m*	5	6	11	1.832	0.677	0.300 to 3.364			13.06	14.85
Zischka (2011)_Rat_Complex I_3.43 m**	3	6	9	-4.108	1.154	-6.838 to -1.379			4.50	11.37
Gu (2000) _Human_Complex I_30-34 y	3	3	6	-3.431	1.185	-6.722 to -0.140			4.26	11.15
Total (fixed effects)	37	42	79	0.544	0.245	0.0567 to 1.032	2.223	0.029	100.00	100.00
Total (random effects)	37	42	79	-0.0163	0.651	-1.312 to 1.279	-0.0250	0.980	100.00	100.00

Test for heterogeneity

Q	38.8397
DF	6
Significance level	$P < 0.0001$
I^2 (inconsistency)	84.55%
95% CI for I^2	69.93 to 92.06

Publication bias

Egger's test	
Intercept	-6.0540
95% CI	-13.0172 to 0.9092
Significance level	P = 0.0757
Begg's test	
Kendall's Tau	-0.1429
Significance level	P = 0.6523

COMPLEX II ACTIVITY

Meta-analysis: continuous measure

Variable for studies	Studies
1. Intervention groups	
Variable for number of cases	n_mut
Variable for mean	mean_mut
Variable for SD	SD_mut
2. Control groups	
Variable for number of cases	n
Variable for mean	mean
Variable for SD	SD

Study	N1	N2	Total	SMD	SE	95% CI	t	P	Weight (%)	
									Fixed	Random
Sauer (2011)_Mouse (KO)_Complex II_0.75 m	7	7	14	0.592	0.513	-0.525 to 1.709			14.20	15.72
Sauer (2011)_Mouse (KO)_Complex II_1.5 m	7	7	14	0.710	0.518	-0.418 to 1.839			13.91	15.67
Sauer (2011)_Mouse (KO)_Complex II_11.75 m	7	7	14	-3.744	0.867	-5.632 to -1.856			4.97	12.30
Medici (2020)_Mouse (KO)_Complex II_6 m	22	22	44	-0.385	0.299	-0.988 to 0.218			41.76	17.44
Zischka (2011)_Rat_Complex II_3 m*	5	6	11	0.000	0.553	-1.252 to 1.252			12.19	15.34
Zischka (2011)_Rat_Complex II_3.3 m*	5	6	11	-0.914	0.587	-2.241 to 0.413			10.84	15.02
Zischka (2011)_Rat_Complex II_3.43 m**	3	6	9	-4.931	1.321	-8.054 to -1.807			2.14	8.52
Total (fixed effects)	56	61	117	-0.369	0.193	-0.751 to 0.0141	-1.908	0.059	100.00	100.00
Total (random effects)	56	61	117	-0.881	0.525	-1.921 to 0.160	-1.677	0.096	100.00	100.00

Test for heterogeneity

Q	36.2584
DF	6
Significance level	P < 0.0001
I^2 (inconsistency)	83.45%
95% CI for I^2	67.40 to 91.60

Publication bias

Egger's test	
Intercept	-3.6045
95% CI	-9.0240 to 1.8150
Significance level	P = 0.1480
Begg's test	
Kendall's Tau	-0.5238
Significance level	P = 0.0985

COMPLEX II-III ACTIVITY

Meta-analysis: continuous measure

Variable for studies	Studies
1. Intervention groups	
Variable for number of cases	n_mut
Variable for mean	mean_mut
Variable for SD	SD_mut
2. Control groups	
Variable for number of cases	n
Variable for mean	mean
Variable for SD	SD

Study	N1	N2	Total	SMD	SE	95% CI	t	P	Weight (%)	
									Fixed	Random
Roberts (2008) _Mouse (tx-j)_Complex II-III_1 m	6	6	12	0.583	0.546	-0.633 to 1.800			19.37	15.72
Roberts (2008) _Mouse (tx-j)_Complex II-III_2 m	6	6	12	-2.262	0.705	-3.833 to -0.691			11.61	14.52
Roberts (2008) _Mouse (tx-j)_Complex II-III_3 m	6	6	12	0.687	0.551	-0.540 to 1.915			19.02	15.68
Roberts (2008) _Mouse (tx-j)_Complex II-III_4 m	6	6	12	0.130	0.533	-1.059 to 1.318			20.29	15.81
Roberts (2008) _Mouse (tx-j)_Complex II-III_5 m	6	6	12	1.315	0.597	-0.0144 to 2.644			16.22	15.35
Roberts (2008) _Mouse (tx-j)_Complex II-III_6 m	6	6	12	2.409	0.725	0.794 to 4.024			10.98	14.36
Gu (2000) _Human_Complex II-III_30-34 y	3	3	6	-4.747	1.517	-8.960 to -0.534			2.51	8.57
Total (fixed effects)	39	39	78	0.366	0.240	-0.112 to 0.845	1.524	0.132	100.00	100.00
Total (random effects)	39	39	78	0.0322	0.615	-1.193 to 1.257	0.0523	0.958	100.00	100.00

Test for heterogeneity

Q	36.4161
DF	6
Significance level	P < 0.0001
I^2 (inconsistency)	83.52%
95% CI for I^2	67.56 to 91.63

Publication bias

Egger's test	
Intercept	-4.8151
95% CI	-13.5926 to 3.9624
Significance level	P = 0.2176
Begg's test	
Kendall's Tau	0.1429
Significance level	P = 0.6523

COMPLEX III ACTIVITY

Meta-analysis: continuous measure

Variable for studies	Studies
1. Intervention groups	
Variable for number of cases	n_mut
Variable for mean	mean_mut
Variable for SD	SD_mut
2. Control groups	
Variable for number of cases	n
Variable for mean	mean
Variable for SD	SD

Study	N1	N2	Total	SMD	SE	95% CI	t	P	Weight (%)	
									Fixed	Random
Sauer (2011)_Mouse (KO)_Complex III_0.75 m	7	7	14	-0.218	0.502	-1.311 to 0.876			34.91	34.91
Sauer (2011)_Mouse (KO)_Complex III_1.5 m	7	7	14	-0.321	0.504	-1.419 to 0.777			34.63	34.63
Sauer (2011)_Mouse (KO)_Complex III_11.75 m	7	7	14	-1.038	0.537	-2.209 to 0.133			30.46	30.46
Total (fixed effects)	21	21	42	-0.503	0.297	-1.103 to 0.0960	-1.697	0.097	100.00	100.00
Total (random effects)	21	21	42	-0.503	0.297	-1.103 to 0.0960	-1.697	0.097	100.00	100.00

Test for heterogeneity

Q	1.4460
DF	2
Significance level	P = 0.4853
I^2 (inconsistency)	0.00%
95% CI for I^2	0.00 to 95.36

Publication bias

Egger's test	
Intercept	-22.4195
95% CI	-42.1704 to -2.6685
Significance level	P = 0.0441
Begg's test	
Kendall's Tau	-1.0000
Significance level	P = 0.1172

COMPLEX IV ACTIVITY

Meta-analysis: continuous measure

Variable for studies	Studies
1. Intervention groups	
Variable for number of cases	n_mut
Variable for mean	mean_mut
Variable for SD	SD_mut
2. Control groups	
Variable for number of cases	n
Variable for mean	mean
Variable for SD	SD

Study	N1	N2	Total	SMD	SE	95% CI	t	P	Weight (%)	
									Fixed	Random
Sauer (2011)_Mouse (KO)_Complex IV_0.75 m	7	7	14	-1.184	0.548	-2.378 to 0.0102			9.53	8.06
Sauer (2011)_Mouse (KO)_Complex IV_1.5 m	7	7	14	-0.936	0.531	-2.092 to 0.220			10.16	8.11
Sauer (2011)_Mouse (KO)_Complex IV_11.75 m	7	7	14	-1.674	0.592	-2.964 to -0.385			8.17	7.95
Medici (2020)_Mouse (KO)_Complex IV_6 m	22	22	44	-9.800	1.086	-11.991 to -7.609			2.43	6.46
Roberts (2008) _Mouse (tx-j)_Complex IV_1 m	6	6	12	1.015	0.572	-0.259 to 2.288			8.76	8.01
Roberts (2008) _Mouse (tx-j)_Complex IV_2 m	6	6	12	0.470	0.541	-0.736 to 1.676			9.77	8.08
Roberts (2008) _Mouse (tx-j)_Complex IV_3 m	6	6	12	-0.866	0.561	-2.117 to 0.385			9.08	8.03
Roberts (2008) _Mouse (tx-j)_Complex IV_4 m	6	6	12	0.549	0.544	-0.664 to 1.762			9.66	8.07
Roberts (2008) _Mouse (tx-j)_Complex IV_5 m	6	6	12	-2.431	0.728	-4.053 to -0.809			5.40	7.57
Roberts (2008) _Mouse (tx-j)_Complex IV_6 m	6	6	12	-1.516	0.616	-2.888 to -0.143			7.54	7.89
Zischka (2011)_Rat_Complex IV_3 m*	5	6	11	0.560	0.566	-0.721 to 1.840			8.93	8.02
Zischka (2011)_Rat_Complex IV_3.3 m*	5	6	11	-0.560	0.566	-1.840 to 0.721			8.93	8.02
Zischka (2011)_Rat_Complex IV_3.43 m**	3	6	9	-4.931	1.321	-8.054 to -1.807			1.64	5.72
Total (fixed effects)	92	97	189	-0.800	0.169	-1.134 to -0.466	-4.728	<0.001	100.00	100.00
Total (random effects)	92	97	189	-1.429	0.534	-2.483 to -0.376	-2.677	0.008	100.00	100.00

Test for heterogeneity

Q	115.2662
DF	12
Significance level	$P < 0.0001$
I^2 (inconsistency)	89.59%
95% CI for I^2	84.05 to 93.21

Publication bias

Egger's test	
Intercept	-11.0576
95% CI	-16.7061 to -5.4090
Significance level	$P = 0.0012$
Begg's test	
Kendall's Tau	-0.4516
Significance level	$P = 0.0316$

COMPLEX V ACTIVITY

Meta-analysis: continuous measure

Variable for studies	Studies
1. Intervention groups	
Variable for number of cases	n_mut
Variable for mean	mean_mut
Variable for SD	SD_mut
2. Control groups	
Variable for number of cases	n
Variable for mean	mean
Variable for SD	SD

Study	N1	N2	Total	SMD	SE	95% CI	t	P	Weight (%)	
									Fixed	Random
Sauer (2011)_Mouse (KO)_Complex V_0.75 m	7	7	14	-0.592	0.513	-1.709 to 0.525			27.09	26.62
Sauer (2011)_Mouse (KO)_Complex V_1.5 m	7	7	14	-0.296	0.503	-1.393 to 0.801			28.09	27.28
Sauer (2011)_Mouse (KO)_Complex V_11.75 m	7	7	14	-0.296	0.503	-1.393 to 0.801			28.09	27.28
Einer (2019)_Rat_Complex V_2.5 m	6	6	12	-1.845	0.652	-3.299 to -0.392			16.72	18.82
Total (fixed effects)	27	27	54	-0.635	0.267	-1.171 to -0.0999	-2.381	0.021	100.00	100.00
Total (random effects)	27	27	54	-0.666	0.324	-1.316 to -0.0172	-2.060	0.044	100.00	100.00

Test for heterogeneity

Q	4.3560
DF	3
Significance level	P = 0.2255
I^2 (inconsistency)	31.13%
95% CI for I^2	0.00 to 75.20

Publication bias

Egger's test	
Intercept	-10.0639
95% CI	-14.9079 to -5.2199
Significance level	P = 0.0123
Begg's test	
Kendall's Tau	-1.0000
Significance level	P = 0.0415

CITRATE SYNTHASE ACTIVITY

Meta-analysis: continuous measure

Variable for studies	Studies
1. Intervention groups	
Variable for number of cases	n_mut
Variable for mean	mean_mut
Variable for SD	SD_mut
2. Control groups	
Variable for number of cases	n
Variable for mean	mean
Variable for SD	SD

Study	N1	N2	Total	SMD	SE	95% CI	t	P	Weight (%)	
									Fixed	Random
Gu (2000) _Human_30-34 y	3	3	6	1.197	0.737	-0.851 to 3.244			9.10	13.44
Medici (2020)_Mouse (KO)_6 m	22	22	44	1.491	0.336	0.812 to 2.169			43.81	14.44
Roberts (2008) _Mouse (tx-j)_1 m	6	6	12	-3.408	0.876	-5.361 to -1.456			6.44	12.98
Roberts (2008) _Mouse (tx-j)_2 m	6	6	12	-7.238	1.571	-10.738 to -3.739			2.01	10.29
Roberts (2008) _Mouse (tx-j)_3 m	6	6	12	-0.327	0.537	-1.524 to 0.869			17.16	14.02
Roberts (2008) _Mouse (tx-j)_4 m	6	6	12	0.262	0.535	-0.931 to 1.455			17.26	14.02
Roberts (2008) _Mouse (tx-j)_5 m	6	6	12	7.601	1.640	3.946 to 11.256			1.84	10.01
Roberts (2008) _Mouse (tx-j)_6 m	6	6	12	6.560	1.441	3.349 to 9.770			2.38	10.80
Total (fixed effects)	61	61	122	0.682	0.222	0.242 to 1.123	3.067	0.003	100.00	100.00
Total (random effects)	61	61	122	0.650	0.918	-1.168 to 2.467	0.708	0.481	100.00	100.00

Test for heterogeneity

Q	92.0716
DF	7
Significance level	P < 0.0001
I^2 (inconsistency)	92.40%
95% CI for I^2	87.38 to 95.42

Publication bias

Egger's test	
Intercept	-0.5263
95% CI	-7.5416 to 6.4890
Significance level	P = 0.8604
Begg's test	
Kendall's Tau	0.0000
Significance level	P = 1.0000

CYTRATE SYNTHASE ACTIVITY-ADULTS ONLY

Meta-analysis: continuous measure

Variable for studies	Studies
1. Intervention groups	
Variable for number of cases	n_mut
Variable for mean	mean_mut
Variable for SD	SD_mut
2. Control groups	
Variable for number of cases	n
Variable for mean	mean
Variable for SD	SD

Study	N1	N2	Total	SMD	SE	95% CI	t	P	Weight (%)	
									Fixed	Random
Gu (2000) _Human_30-34 y	3	3	6	1.197	0.737	-0.851 to 3.244			12.23	22.00
Medici (2020)_Mouse (KO)_6 m	22	22	44	1.491	0.336	0.812 to 2.169			58.90	24.89
Roberts (2008) _Mouse (tx-j)_4 m	6	6	12	0.262	0.535	-0.931 to 1.455			23.20	23.64
Roberts (2008) _Mouse (tx-j)_5 m	6	6	12	7.601	1.640	3.946 to 11.256			2.47	13.92
Roberts (2008) _Mouse (tx-j)_6 m	6	6	12	6.560	1.441	3.349 to 9.770			3.20	15.55
Total (fixed effects)	43	43	86	1.483	0.258	0.970 to 1.996	5.750	<0.001	100.00	100.00
Total (random effects)	43	43	86	2.775	0.902	0.980 to 4.569	3.075	0.003	100.00	100.00

Test for heterogeneity

Q	31.6729
DF	4
Significance level	$P < 0.0001$
I^2 (inconsistency)	87.37%
95% CI for I^2	72.91 to 94.11

Publication bias

Egger's test	
Intercept	3.6678
95% CI	-2.6348 to 9.9705
Significance level	$P = 0.1611$
Begg's test	
Kendall's Tau	0.6000
Significance level	$P = 0.1416$

MITOCHONDRIAL MORPHOLOGY

Meta-analysis: continuous measure

Variable for studies	Study_number<br>Study number
1. Intervention groups	
Variable for number of cases	n_mut<br>n-mut
Variable for mean	mean_mut<br>mean-mut
Variable for SD	SD_mut<br>SD-mut
2. Control groups	
Variable for number of cases	n
Variable for mean	mean
Variable for SD	SD

Study	N1	N2	Total	SMD	SE	95% CI	t	P	Weight (%)	
									Fixed	Random
Einer (2019)_Rat_Aberrant	6	6	12	0.923	0.565	-0.336 to 2.182			72.56	31.16
Lichtmannegger (2016)_Rat_Aberrant*	3	3	6	8.381	2.506	1.425 to 15.338			3.69	17.60
Lichtmannegger (2016)_Rat_Aberrant**	3	3	6	4.787	1.528	0.545 to 9.029			9.93	24.72
Lichtmannegger (2016)_Rat_Aberrant***	5	3	8	4.514	1.294	1.346 to 7.681			13.83	26.51
Total (fixed effects)	17	15	32	2.078	0.481	1.095 to 3.061	4.317	<0.001	100.00	100.00
Total (random effects)	17	15	32	4.143	1.553	0.972 to 7.315	2.668	0.012	100.00	100.00

Test for heterogeneity

Q	17.1929
DF	3
Significance level	P = 0.0006
I^2 (inconsistency)	82.55%
95% CI for I^2	55.19 to 93.21

Publication bias

Egger's test	
Intercept	4.1080
95% CI	2.6096 to 5.6064
Significance level	P = 0.0071
Begg's test	
Kendall's Tau	0.6667
Significance level	P = 0.1742

OXIDATIVE STRESS-PART 1

Meta-analysis: continuous measure

Variable for studies	Study_number<br>Study number
1. Intervention groups	
Variable for number of cases	n_mut<br>n-mut
Variable for mean	mean_mut<br>mean-mut
Variable for SD	SD_mut<br>SD-mut
2. Control groups	
Variable for number of cases	n
Variable for mean	mean
Variable for SD	SD

Study	N1	N2	Total	SMD	SE	95% CI	t	P	Weight (%)	
									Fixed	Random
Sokol (1994)_Dog_Lipid conjugated diene_	4	4	8	2.988	0.967	0.622 to 5.355			7.21	14.59
Sokol (1994)_Human_Lipid conjugated diene_25 y	3	8	11	9.149	2.046	4.520 to 13.779			1.61	9.08
Einer (2019)_Rat_mtROS_2.5 m	6	6	12	0.000	0.533	-1.187 to 1.187			23.77	16.61
Medici (2020)_Mouse_mtROS_6 m	22	22	44	3.081	0.442	2.189 to 3.974			34.49	16.92
Einer (2019)_Rat_mtROS_2.5 m	6	6	12	0.000	0.533	-1.187 to 1.187			23.77	16.61
Sokol (1994)_Dog_TBARS_	4	4	8	5.038	1.401	1.609 to 8.467			3.44	12.24
Sokol (1994)_Human_TBARS_25 y	3	8	11	4.186	1.086	1.729 to 6.642			5.72	13.95
Total (fixed effects)	48	58	106	1.838	0.260	1.323 to 2.353	7.078	<0.001	100.00	100.00
Total (random effects)	48	58	106	2.989	0.885	1.235 to 4.743	3.379	0.001	100.00	100.00

Test for heterogeneity

Q	55.7801
DF	6
Significance level	P < 0.0001
I^2 (inconsistency)	89.24%
95% CI for I^2	80.34 to 94.12

Publication bias

Egger's test	
Intercept	3.8631
95% CI	-2.1303 to 9.8564
Significance level	P = 0.1584
Begg's test	
Kendall's Tau	0.5000
Significance level	P = 0.1148

OXIDATIVE STRESS-PART 2

Meta-analysis: continuous measure

Variable for studies	Study_number<br>Study number
1. Intervention groups	
Variable for number of cases	n_mut<br>n-mut
Variable for mean	mean_mut<br>mean-mut
Variable for SD	SD_mut<br>SD-mut
2. Control groups	
Variable for number of cases	n
Variable for mean	mean
Variable for SD	SD

Study	N1	N2	Total	SMD	SE	95% CI	t	P	Weight (%)	
									Fixed	Random
Sauer (2011)_Mouse_Aconitase_0.75 m	7	7	14	0.592	0.513	-0.525 to 1.709			11.97	8.35
Sauer (2011)_Mouse_Aconitase_1.5 m	7	7	14	2.664	0.710	1.117 to 4.210			6.24	7.75
Sauer (2011)_Mouse_Aconitase_12 m	7	7	14	1.588	0.583	0.317 to 2.860			9.24	8.15
Zischka (2011)_Rat_Aconitase_2 m	3	4	7	2.388	0.905	0.0605 to 4.715			3.84	7.08
Zischka (2011)_Rat_Aconitase_3 m	5	4	9	1.110	0.651	-0.429 to 2.649			7.43	7.94
Zischka (2011)_Rat_Aconitase_4 m*	2	4	6	-3.482	1.220	-6.869 to -0.0955			2.11	5.98
Nagasaka (2006)_Human_MnSOD_3-12 y	13	12	25	-2.245	0.501	-3.280 to -1.209			12.55	8.38
Sauer (2011)_Mouse_MnSOD_0.75 m	7	7	14	3.552	0.837	1.728 to 5.376			4.49	7.32
Sauer (2011)_Mouse_MnSOD_1.5 m	7	7	14	0.000	0.500	-1.090 to 1.090			12.57	8.39
Sauer (2011)_Mouse_MnSOD_12 m	7	7	14	0.000	0.500	-1.090 to 1.090			12.57	8.39
Ohhira (1995)_Rat_MnSOD_2 m	4	4	8	-0.207	0.616	-1.716 to 1.301			8.28	8.05
Ohhira (1995)_Rat_MnSOD_4 m	4	4	8	-1.583	0.731	-3.371 to 0.205			5.89	7.68
Ohhira (1995)_Rat_MnSOD_5 m	4	4	8	-3.429	1.055	-6.010 to -0.849			2.83	6.55
Total (fixed effects)	77	78	155	0.155	0.177	-0.196 to 0.505	0.872	0.384	100.00	100.00
Total (random effects)	77	78	155	0.143	0.509	-0.862 to 1.147	0.281	0.779	100.00	100.00

Test for heterogeneity

Q	93.5734
DF	12
Significance level	P < 0.0001
I^2 (inconsistency)	87.18%
95% CI for I^2	79.83 to 91.85

Publication bias

Egger's test	
Intercept	0.09627
95% CI	-6.9137 to 7.1062
Significance level	P = 0.9764
Begg's test	
Kendall's Tau	0.03896
Significance level	P = 0.8529

mtDNA COPY NUMBER

Meta-analysis: continuous measure

Variable for studies	Studies
1. Intervention groups	
Variable for number of cases	n_mut<br>n-mut
Variable for mean	mean_mut<br>mean-mut
Variable for SD	sd_mut<br>sd-mut
2. Control groups	
Variable for number of cases	n
Variable for mean	mean
Variable for SD	sd

Study	N1	N2	Total	SMD	SE	95% CI	t	P	Weight (%)	
									Fixed	Random
Medici (2020)_Human_PBMC_36 y	47	37	84	-1.486	0.246	-1.976 to -0.997			49.33	27.94
Medici (2020)_Mouse (KO)_Liver_6 m	22	22	44	-1.300	0.327	-1.960 to -0.641			27.95	25.18
Roberts (2008) _Mouse (tx-j)_Liver_2 m	6	3	9	0.000	0.628	-1.485 to 1.485			7.57	15.63
Roberts (2008) _Mouse (tx-j)_Liver_5 m	6	3	9	0.000	0.628	-1.485 to 1.485			7.57	15.63
Roberts (2008) _Mouse (tx-j)_Liver_6 m	6	3	9	0.000	0.628	-1.485 to 1.485			7.57	15.63
Total (fixed effects)	87	68	155	-1.097	0.173	-1.438 to -0.755	-6.344	<0.001	100.00	100.00
Total (random effects)	87	68	155	-0.743	0.344	-1.422 to -0.0629	-2.158	0.032	100.00	100.00

Test for heterogeneity

Q	12.0407
DF	4
Significance level	P = 0.0171
I^2 (inconsistency)	66.78%
95% CI for I^2	13.66 to 87.22

Publication bias

Egger's test	
Intercept	3.9358
95% CI	3.2415 to 4.6302
Significance level	P = 0.0004
Begg's test	
Kendall's Tau	1.0000
Significance level	P = 0.0143
